# Effect of Tamoxifen on Proteome Expression during *In Vitro* Myogenesis in Murine Skeletal Muscle C_2_C_12_ Cells

**DOI:** 10.1021/acs.jproteome.3c00340

**Published:** 2023-08-08

**Authors:** Emily A. Morris, Ahlenne Abreu, Stylianos P. Scordilis

**Affiliations:** † Department of Microbiology and Immunology, Geisel School of Medicine at Dartmouth, Borwell Building 644E, Lebanon, New Hampshire 03756, United States; ‡ Department of Cancer Biology, Perelman School of Medicine, University of Pennsylvania Medical School, 421 Curie Blvd. Room 612 BRB II/III, Philadelphia, Pennsylvania 19104, United States; § Department of Biological Sciences, Smith College, Ford Hall 202 B, Northampton, Massachusetts 01063, United States

**Keywords:** tamoxifen, myogenesis, functional proteomics, TMT-multiplex, metallothionein 1, adiponectin signaling

## Abstract

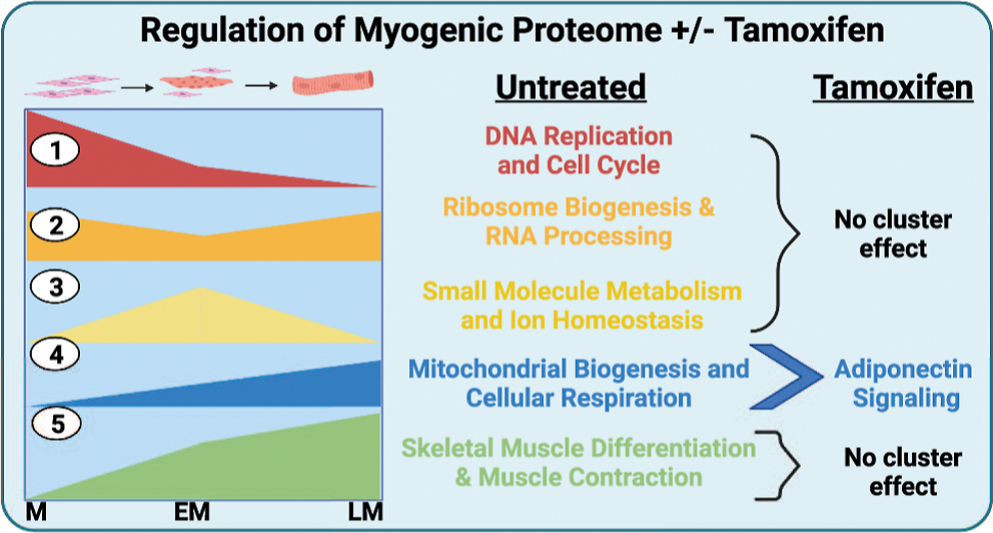

Tamoxifen (TMX), a selective estrogen receptor modulator, is commonly used in the treatment of hormone-responsive cancers. However, the effects of TMX in anabolic tissues harboring estrogen receptors, such as skeletal muscle, are poorly understood. We report a tandem mass-tag approach to TMX-treated myogenesis in C_2_C_12_ cells, a well-characterized model of *in vitro* murine skeletal muscle differentiation. A longitudinal analysis of >10,000 proteins identified in untreated C_2_C_12_ myogenesis revealed a novel subset of 1,062 myogenically regulated proteins. These proteins clustered into five distinct longitudinal expression trends which significantly overlap those obtained in similar analyses performed in human myocytes. We document a specific functional enrichment for adiponectin-signaling unique to TMX-treated myogenesis, as well as a subset of 198 proteins that are differentially expressed in TMX-treated cells relative to controls at one or more stages of myogenesis, the majority of which were involved in steroid and lipid metabolism. Further analysis highlights metallothionein-1 as a novel target of TMX treatment at each stage of C_2_C_12_ myogenesis. Finally, we present a powerful, self-validating pipeline for analyzing the total proteomic response to *in vitro* treatment across every stage of muscle cell development which can be easily adapted to study the effects of other drugs on myogenesis.

## 1 Introduction

Myogenesis occurs both during embryonic development and after eccentric damage to existing muscle tissue. In adult skeletal muscle, myoblasts are the mononucleated muscle progenitor/stem cells (satellite cells) which remain in pluripotent quiescence between the sarcolemma and basal lamina and are responsible for the formation of new muscle fibers. Plasmalemmal damage resulting from eccentric exercise releases myokines that signal these satellite cells to move into the damaged tissue and begin asymmetric proliferation. When the myoblasts are fully confluent in the site of the tear, they align with each other to form cell–cell junctions *via* M-cadherin, and depolarization due to calcium influx signals the cells to differentiate and begin fusing into multinucleated early myotubes.^1,2^ This differentiation process results in permanent cell cycle exit and activation of a muscle tissue-specific program of gene and protein expression.^3^ The multinucleated myotubes devote resources to the synthesis of mitochondrial and contractile protein apparati, and late myotubes contain some functional sarcomeres capable of contraction and neuromuscular response.^4^ Myoblasts, early, and late myotubes express varying levels of estrogen receptors (ERs, ERα, ERβ, and GPER), and their proliferative, hypertrophic, and contractile activities are also known to be regulated by androgen signaling.^5^

While transcriptomic studies are useful, multiple studies have shown that mRNA transcription levels do not always correlate to corresponding protein levels. In addition, the effects of a transcription modulator such as estrogen on cellular function cannot be fully understood without a proteomic analysis.^50^ A proteomic data set with deep coverage provides novel insights into the potential off-target effects of many drugs. Here, we focus on the effects of tamoxifen (TMX), one of the most widely used treatments for breast cancer.

One in eight women will develop breast cancer in their lifetimes, and breast cancer is the second leading cause of cancer-related death for women living in the U.S.^6^ Two out of every three breast cancer cases are classified as hormone receptor positive, and as such, these neoplasms are dependent on estrogen for growth.^7^ TMX is a selective estrogen receptor modulator (SERM) and a well-established treatment for ER-positive breast cancer. SERMs bind competitively to ERs in androgenic tumor tissue and prevent estrogen from functioning in its role as a transcription factor in these cells.^8^ While this is an effective therapeutic approach to inhibiting tumor growth in many breast cancers, ERs are not confined to androgenic tissue. Anabolic tissues, such as skeletal muscle, also express ERs, and the development and repair of skeletal muscle tissue (myogenesis) are dependent on ER expression, estrogen-mediated gene transcription, and the changes in protein expression that occur.^9,10^ However, the downstream biochemical effects of SERMs like TMX on developing muscle tissue, particularly at the proteomic level, are unknown.

We therefore sought to perform a foundational proteomic experiment into the effect of TMX on early muscle development. Although primary human myocytes are a preferred model when available, C_2_C_12_ cells (immortalized mouse-derived skeletal muscle stem cells) are a well-studied alternative model of *in vitro* muscle development and repair. C_2_C_12_ cells, which can be triggered to differentiate in a controlled manner in culture, can be used to study the effects of drug treatments (not requiring liver or other organ-based metabolism for activation) on developing muscle tissue.^11^ While the proteome of developing C_2_C_12_ cells has been analyzed in the past, newer proteomic methods, such as tandem mass-tag (TMT), have since been developed that allow for high-throughput, quantitative multiplexing of multiple samples, and treatment conditions.^12−15^

In this study, we applied a TMT-based approach to TMX-treated C_2_C_12_ myogenesis. We first conducted a longitudinal analysis of >10,000 proteins identified across untreated C_2_C_12_ myogenesis, which revealed a novel subset of 1,062 myogenically regulated proteins. This set of regulatory proteins had significant (homologous) overlap with similar analyses performed in human myocytes and clustered into five distinct longitudinal expression trends. We compared a longitudinal analysis of TMX-treated myogenesis to untreated myogenesis and found that the vast majority of myogenically regulated proteins were unaffected by TMX treatment and followed the same expression trends when compared to controls. However, when analyzing differential protein expression at each individual stage of myogenesis, we identified a set of 198 proteins that were differentially expressed (DE) in TMX-treated cells relative to controls. Further analysis revealed a subset of 30 proteins that were also regulated during normal myogenesis. GO enrichment revealed a specific effect of TMX on proteins involved in steroid biosynthesis, lipid storage, and metal ion homeostasis. The primary proteomic effect of TMX was in myoblasts (pluripotent cells) and late myotubes (well differentiated cells), with minimal effect on the proteome of early myotubes. From this subset, we also identified 10 proteins that were most significantly regulated by TMX during myogenesis (FC ≥ 2 at least two out of three time points). Four of these proteins are known downstream effectors of TMX in non-muscle models, while the remaining six have not previously been identified as TMX-regulated proteins. Finally, we present a powerful, self-validating pipeline for analyzing the total proteomic response to *in vitro* treatment across every stage of muscle cell development which can be used to study the effect of other drugs in the future.

## 2 Materials and Methods

### 2.1 C_2_C_12_ Cell Culture

Frozen subclones of the cell line were obtained from the American Tissue Culture Collection (ATCC, #CRL-1772) and thawed/resuspended in 10% FBS/DMEM (10% fetal bovine serum Cat. #: 26140-079, 0.5% penicillin/streptomycin, 89.5% Dulbecco’s modified Eagle’s medium supplemented with 4.5 g/L glucose and l-glutamine Cat. #: 11965-092, Gibco). Aspirated cells were split evenly across four 75 cm^2^ flasks (Cat. #: 353136, BD Falcon). Cells were allowed to grow for 2 days (until approximately 80% confluent) at 37 °C in a humidified incubator supplemented with 5% CO_2_ and were observed by phase contrast microscopy every 24 h.

Confluent cells were combined and split into 45 separate 25 cm^2^ culture flasks to create the following nine experimental conditions in quintuplicate: control media (CTRL) 10% FBS/DMEM myoblast, early and late myotube flasks [5% horse serum (HS) Lot #: 1517703, DMEM]; vehicle control media (EtOH) (10% FBS/DMEM Gibco) myoblast, early and late myotube flasks 5% HS/DMEM; and TMX (Cayman Chemical Cat. #: 10540-29-1) (10% FBS/DMEM, 0.2 μM TMX in EtOH) myoblast, and early and late myotube flasks (5% HS/DMEM). The 25 cm^2^ flasks were seeded at a concentration of 10^5^ cells, and conditional media were changed every 72 h. Cells were checked every 24 h until 80% confluency was reached.

When the myoblast flasks reached 80% confluency, cells were stepped down to induce differentiation. Step down was performed by changing the 10% FBS conditional media to 5% HS/DMEM conditional media to initiate fusion between myoblasts to form myotubes for 30 out of 45 flasks (10 flasks per experimental condition with the appropriate media; CTRL, EtOH, or TMX); the other 15 flasks of myoblasts (5x per media conditions) were harvested at 80% confluency as the “Day 0” time point. Harvesting was performed by trypsinizing cells, transferring to a 15 mL conical tube, centrifuging at 600*g* for 5 min. Cells were rinsed once with PBS (DPBS, Cat. #: 14190-136, Gibco) and resuspended in 250 μL of 1X SDS sample buffer (Laemmli, 1970). Resuspensions were placed in sterile 1.5 mL microfuge tubes and heated at 100 °C for 5 min to denature proteins. Samples were then homogenized using a polytron (PT 1200 C, Polytron Kinematica Tissue Homogenizer, setting 4) in three 15 s bursts and frozen at −20 °C until a Lowry assay was performed to estimate the total protein concentration. The remaining cells were fed every 3 days and observed by phase contrast microscopy every 24 h. Extraction and protein estimation were repeated as described above on day 5 (early myotube stage; *n* = 5x per medium condition; resuspended in 350 μL of 1X SDS sample buffer) and again on day 9 (late myotube stage; *n* = 5x per medium condition; resuspended in 450 μL of 1X SDS sample buffer).

### 2.2 Dose Response

To visualize morphological changes that occur in response to growing in TMX, a dose–response assay was performed at four different concentrations of TMX (0.1, 1, 10, and 100 μM).

Subclones of the C_2_C_12_ cell line were split into 45 separate 25 cm^2^ culture flasks to create the following 15 experimental conditions in triplicate: CTRL medium (10% FBS/DMEM) myoblast, early and late myotube flasks (5% HS/DMEM); four different concentrations of TMX in EtOH (0.1, 1, 10, and 100 μM) in 10% FBS/DMEM myoblast, and in early and late myotube flasks (5% HS/DMEM). Cells grown in 100 μM TMX were non-viable after day 0, so flasks containing 100 μM TMX were not stepped down. The 25 cm^2^ flasks were seeded at a concentration of 10^5^, and condition media were changed every 3 days; the cells were checked every 24 h until 80% confluency was reached. The cells were allowed to grow in a 37 °C humidified incubator, supplemented with 5% CO_2_. Cells were stepped-down as described above after 80% confluency was reached and harvested at the three time points of development (see C_2_C_12_ Cell Culture section).

### 2.3 Total Protein Estimation

Total protein concentrations from C_**2**_C_**12**_ samples were estimated using a modified Lowry assay by trichloroacetic acid precipitation.^16^ The samples were assayed in triplicate with a bovine serum albumin (BSA) (5–120 μg) standard curve. All tubes were brought to a volume of 0.8 mL with RO H_**2**_O and treated with 0.2 mL of 50% TCA to precipitate the protein. The tubes were then vortexed and centrifuged at 4 °C, 20,000 rpm for 15 min. After centrifugation, the supernatant was immediately removed, and the pellet was treated with 0.25 mL of 1 N NaOH and 0.25 mL of RO H_**2**_O and vortexed. Next, 2.5 mL of 0.5% CuSO_**4**_/1% Na-K tartrate in 2% Na_**2**_CO_**3**_ with a 1:50 (v/v) ratio was added and vortexed. After sitting for 10 min, 0.25 mL of the diluted Folin-Ciocalteu phenol reagent (1:1 in RO H_**2**_O) was added to each tube and vortexed. The reaction was allowed for 30 min, and then, absorbances were measured using a PerkinElmer Lambda 25 UV/Vis spectrophotometer at 750 nm, with RO H_**2**_O to blank the instrument. Lastly, KaleidaGraph (Synergy, Version 4.5.4) software was used to obtain the BSA standard curve and subsequently determine the concentrations of individual samples.

### 2.4 Sodium Dodecyl Sulfate-Polyacrylamide Gel Electrophoresis (SDS-PAGE)

Proteins were separated based on their apparent molecular weight using a modified version of the Laemmli SDS-PAGE^17^ with a 5% for stacking and 10% separating gel. 10 μg of protein from each sample was loaded alongside a molecular-weight standard (Precision Plus Protein Kaleidoscope, Bio-Rad). Gels were run at 94 V and were stopped when the dye front reached ∼1 cm from the bottom.

Following electrophoresis, the separating gel was diffusion stained (45.5% methanol, 45.5% RO H_2_O, 9.0% acetic acid, and 0.25% Coomassie Brilliant Blue R250) and gently agitated overnight. The next day, the gel was destained twice with destain 1 (45.5% methanol, 45.5% RO H_2_O, and 9.0% acetic acid) in 3 h intervals with agitation. Then, the gel was placed in destain 2 overnight and agitated. Finally, the gel was stored in 7.5% acetic acid for 1 day and scanned the following day with an Epson Expression 1680 Scanner (Version 3.04A, Professional Mode, Reflective, Photo Auto Exposure type, 24-bit color and 8-bit gray-scale, Best Scanning Quality, dpi 1200, gamma 1.58).

### 2.5 Colorimetric Immunoblotting for Confirmation of Myoblast Differentiation

After a gel finished running, it was placed in cold transfer buffer (192 mM glycine, 25 mM Tris, and 15% v/v methanol) for 15 min prior to transfer to a poly(vinylidene difluoride) (PVDF) membrane at 4 °C for 2 h at 50 V in a modification of Towbin.^18^

After the transfer was completed, the gel was stained with Coomassie Brilliant blue R250 to ensure that the transfer was efficient. The PVDF membrane was transferred to a hybridization tube and blocked with 5 mL of Tris-buffered saline (TBS) (150 mM NaCl, 10 mM Tris–HCl, pH 7.5) supplemented with 5% powdered non-fat dry milk for 2 h at RT on a rotisserie, the blot was rinsed with TBS with 0.05% Tween-20 (TBS-T) for 10 min. Following the wash, 5 mL of an appropriately diluted primary antibody solution was added, and the blot was incubated in this solution of TBS-T with 1.0% BSA fraction V (Sigma-Aldrich, 85040C) at 4 °C overnight in a rotisserie (see Table 1 for primary antibody dilutions used).

**Table 1:** Dilutions of Primary Antibody

antibody name	antibody type (host)	manufacturer	dilution
alpha actin	Polyclonal IgG (rabbit)	ProteinTech, 23660-1	1:2500
androgen receptor	Polyclonal IgG (rabbit)	ProteinTech, 22089-1	1:2500
annexin A1	Monoclonal IgG1 (mouse)	ProteinTech, 66344-1	1:2500
estrogen receptor alpha	Polyclonal IgG (rabbit)	ProteinTech, 21244-1	1:2500
estrogen receptor beta	Polyclonal IgG (rabbit)	Invitrogen, PA1-310B	1:2500
citrate synthase	Polyclonal IgG (rabbit)	ProteinTech, 16131-1	1:2500
creatine kinase-M	Monoclonal IgG1 (mouse)	SantaCruz, 365046	1:2500
GAPDH	Monoclonal IgG2b (mouse)	ProteinTech, 60004-1	1:25,000
G-protein-coupled estrogen receptor	Polyclonal IgG (rabbit)	Invitrogen, PA5-28647	1:2500
superoxide dismutase 1	Monoclonal IgG2a (mouse)	ProteinTech, 67480-1	1:7500
superoxide dismutase 2	Monoclonal IgG2a (mouse)	ProteinTech, 66474-1	1:25,000

The next day, the blot was washed five times with 5 mL of TBS-T for 5 min each. Following the washes, 5 mL of a secondary antibody solution of either an anti-mouse HRP-coupled antibody or an anti-rabbit HRP-coupled antibody depending on the primary antibody were added (Table 2). These antibody solutions in BSA were incubated at room temperature for 2 h. Then, the blot was washed five times with 5 mL of TBS-T for 5 min each and then twice with TBS for 5 min each. The blot was developed with TMB peroxidase (KPL, 50-77-00) until bands were visible and immediately placed in ultrapure water for 5 min before drying overnight between two pieces of Whatman paper. Dry blots were imaged with an Epson Expression 1680 Scanner (Version 3.04A, Professional Mode, Reflective, Photo Auto Exposure type, 24-bit color, Best Scanning Quality, dpi 1200, gamma 1.58).

**Table 2:** Dilutions of Secondary Antibody

antibody name	manufacturer	dilution
goat anti-mouse IgG HRP conjugate	ProteinTech, SA00001-1	1:5,000, 1:3000
goat anti-rabbit IgG HRP conjugate	Cell Signaling, 7074P2	1:5000

### 2.6 Chemiluminescent Immunoblotting

For representative creatine kinase-M blots, 20 μg of protein (Lowry assay^16^) were loaded onto a 10% SDS gel from *n* = 3 replicates per time point from the CTRL (untreated) myogenesis samples and *n* = 3 replicates per time point from the TMX-treated myogenesis; replicates were identical to those used for downstream TMT analysis. Gels were run at 130 V for 85 min at room temperature and transferred to nitrocellulose membranes at 100 V for 60 min. Nitrocellulose blots were Ponceau stained for 5 min and washed with ddH_2_O to remove excess stain prior to colorimetric imaging. Imaged blots were then washed with TBS-T several times to remove the remaining Ponceau stain, then transferred to cassettes, and blocked (5% nonfat dry milk in TBS-T) for 1 h at RT. Blocking buffer was removed prior to addition of primary antibody (diluted in 5% nonfat dry milk + 0.2% azide) and incubated overnight at 4 °C. After incubation, primary antibody solution was removed, and blots were washed 5 X in ddH_2_O and 2 X in TBS-T before secondary HRP-coupled antibody (diluted in 5% milk) was added. Blots were incubated in secondary antibody for 1 h at RT, washed again as described above, and imaged using chemiluminescence. All images were generated using a ChemiDOC MP (Bio-Rad).

### 2.7 Tryptic Digestion

The S-TRAP protocol was employed to carry out the tryptic digestion of all 45 samples (Protofi.com; C02-mini-40). Based on the results from the Lowry assay,^16^ 200 μg of protein from each sample were placed in a clean microfuge tube. Adding DL-1,4-dithiothreitol (#B0142350, Acros-Organics) to a final concentration of 20 mM reduced the disulfide bonds as they were heated to 95 °C at 300 rpm for 9 min in a thermal mixer (Mixer HC, USA Scientific). The samples were allowed to cool to ambient temperature and then irreversibly alkylated by adding iodoacetamide (#UC276366, single use, 9.3 mg per tube, Thermo Scientific) to a final concentration of 40 mM. The samples were incubated in the dark for 30 min at room temperature.

After incubation, samples were centrifuged for 8 min at 13,100*g* at 4 °C, and aqueous phosphoric acid was added to each microfuge tube to a final concentration of 1.2% and vortexed. Next, S-TRAP binding buffer was made of 90% aqueous methanol (#189397, Optima LC/MS, Fisher Chemical) and a final concentration of 100 mM triethylammonium bicarbonate (TEAB, #UD282109A) at pH 7.1 (for starting 50 μL volumes, a ratio 1:7 v/v of sample to S-TRAP binding buffer was maintained). Binding buffer (861.7 μL) was added to each sample and vortexed. The acidified protein solution was transferred from the microfuge tube to an S-TRAP mini column (C01-mini-80, Protofi) and attached to a 2 mL receiver tube with a maximum loading volume of 450 μL. Between each loading, the tubes were centrifuged (Eppendorf Centrifuge 5417R) at 4000*g* and 4 °C for 1 min to allow the binding buffer to flow through the S-TRAP column. The flow-through was disposed of in a hazardous waste container.

After all of the protein solution was transferred from the microfuge tube to the column, the column was washed three times with 400 μL of binding buffer. Then, the column was moved to a new 2 mL receiver tube where the protease could be introduced to the samples. Trypsin Protease MS-Grade (#UH287761, Pierce) was added to each column by diluting 4 μg of trypsin in 125 μL of digestion buffer (50 mM TEAB) per 200 μg of protein. The tubes were sealed with parafilm and incubated overnight at 37 °C in a humidified environment.

The following morning, the peptides were eluted by adding 80 μL of digestion buffer to each spin column. Samples were centrifuged for 1 min at 1000*g*. Next, 80 μL of 0.2% aqueous formic acid were added to the spin columns, and they were centrifuged at 1000*g* for 1 min again. Lastly, hydrophobic peptides were eluted by the addition of 80 μL of acetonitrile (ACN, #013349, HPLC Grade, Fisher Chemicals) containing 0.2% formic acid and centrifuged at 4000*g* for 1 min. The resulting elution was transferred to a clean microfuge tube and lyophilized (Centrivap Concentrator, Labconco). The dried peptides were resuspended in 150 μL of 100 mM TEAB for subsequent analyses.

### 2.8 Fluorometric Peptide Quantification Assay

Peptide concentrations from the S-TRAP were estimated using the quantitative fluorescence peptide assay (#UJ289782, Thermo Scientific Pierce). A nine-point standard curve (0–1000 μg/mL) was created *via* serial dilution of the peptide digest assay standard stock (1 mg/mL) and run in triplicate. To maintain the sample: assay buffer: assay reagent ratio of 1:7:2 (v/v), the volumes of the standards were adjusted to contain 100 mM TEAB similar to the samples. A 96-well microplate (#E18103LE, Black, Greiner Bio-One) was used to plate the blank, standards, samples, and assay reagents. Specifically, the blank wells (A4-6) contained 10 μL of RO H_2_O and 5 μL of 100 mM TEAB; the standard wells (A1-H3) contained 10 μL of the appropriate standard and 5 μL of 100 mM TEAB; lastly, the sample wells (B3-H9) contained 5 μL of the sample and 10 μL of RO H_2_O. All of the wells quickly received 105 μL of fluorometric peptide assay buffer and 30 μL of the fluorometric peptide assay reagent.

The plate was sealed with parafilm, covered with aluminum foil, and incubated for 5 min at ambient temperature. The fluorescence of each well was measured using a Spectramax MS Multi-Detection Microplate Reader (Software: SoftMaxPro 7.0) at 390 and 475 nm. Finally, the standard curve was generated using KaleidaGraph software (Synergy, Version 4.5.4) to calculate the peptide concentrations of all 45 samples.

### 2.9 Isobaric Mass Tagging

Of the total 45 samples, 27 samples (*n* = 3) were used for TMT-labeling and subsequent statistical analyses. 40 μg from each sample were resuspended in 40 μL of 166 mM EPPS buffer (E9502, Sigma-Aldrich), pH 8.5. A representative bridge channel was also generated by taking 15 μg from *n* = 1 replicate per time point per treatment condition (135 μg total), combined into one tube, resuspending in 135 μL of 166 mM EPPS pH 8.5, and dividing into 3 × 40 μL aliquots (15 μL leftover). TMT10plex^TM^ Label Reagent Set (#UL296796, Thermo Fisher Scientific) was equilibrated to room temperature, to avoid moisture condensation. Each vial was reconstituted in 80 μL of anhydrous ACN (#132079, Fisher Scientific), and 3 μL of the reagent were added to each sample (see Figure S1E for TMT tagging scheme and multiplex layout), vortexed, and allowed to react at room temperature for 1 h. The labeling efficiency was checked by combining 2 μL of each reaction with 2 μL of 5% hydroxylamine in EPPS buffer to quench for 20 min at RT (remaining reactions were frozen at −80°). Labeling checks were acidified with 20% TFA to pH 3 and desalted on C18 STAGE tips prior to the LC-MS/MS injection and confirmed by including the TMT label as a dynamic modification in the subsequent searches to ensure a labeling efficiency > 95%. Fully labeled reactions were thawed and quenched with 4 μL of 5% hydroxylamine in EPPS for 20 min at RT, and quenched reactions were acidified with 3x volumes of 1% TFA (final pH = 3), combined into their respective TMT 10-plexes, and desalted on a 10 mg OASIS desalting plate (Waters). Half of each desalted multiplex was offline-fractionated by pentafluorophosphate (PFP) RP-HPLC into 48 fractions as previously described and concatenated into 16 final fractions prior to the LC-MS/MS injection.^19^

### 2.10 LC-MS/MS Analyses

LC-MS/MS analysis was performed on an Orbitrap Fusion Tribrid mass spectrometer (Thermo Fisher Scientific, San Jose, CA) equipped with EASY-nLC 1000 ultra-high pressure liquid chromatography (Thermo Fisher Scientific, Waltham, MA). Peptides were dissolved in loading buffer [5% methanol (Fisher)/1.5% formic acid] and injected directly onto an in-house pulled, polymer coated, fritless, fused silica analytical resolving column (32 cm length, 100 μm inner diameter; PolyMicro) packed with ReproSil, C18 AQ 1.9 μm 120 Å pore stationary phase particles. Peptides in 1.5 μL loading buffer were loaded at 450 bar by chasing on to the column with 8 μL of loading buffer. Samples were separated with a 120 min gradient of 4–33% LC-MS buffer B (LC-MS buffer A: 0.125% formic acid, 3% ACN; LC-MS buffer B: 0.125% formic acid, 95% ACN) at a flow rate of 330 nL/min. The MS was operated with an Orbitrap MS1 scan at 120 K resolution and an AGC target value of 500 K. The maximum injection time was 100 milliseconds, the scan range was 350 to 1500 *m*/*z*, and the dynamic exclusion window was 15 s (±15 ppm from precursor ion *m*/*z*). Precursor ions were selected for MS2 using quadrupole isolation (0.7 *m*/*z* isolation width) in a “top speed” (2 s duty cycle), data-dependent manner. MS2 scans were generated through collision-induced dissociation (CID) fragmentation (35% CID energy) and linear ion trap analysis (Rapid setting). Ion charge states of +2 through +4 were selected for CID MS2. The MS2 scan maximum injection time was 60 milliseconds, and the AGC target value was 60 K. For TMT quantification, the top 8 MS2 peaks were dynamically isolated and further fragmented by higher-collision energy at 55% *via* SPS-MS3 for quantification of liberated reporter ions (110–500 *m*/*z*) in the Orbitrap at 50 K resolution.

### 2.11 Peptide Spectral Matching and Bioinformatics

Raw data were searched using COMET against a target-decoy version of the mouse (*Mus musculus*) proteome sequence database (Uniprot; downloaded 2019; 17,029 fully validated proteins) with a precursor mass tolerance of ±1.00 Da and requiring fully tryptic peptides with up to three missed cleavages, carbamidomethyl cysteine as a fixed modification, and oxidized methionine as a variable modification.^53^ The mass of the TMT reagent (229.162932 Da) was added as a static modification to all peptide N-termini and lysine residues. The resulting peptide spectral matches were filtered to ≤1% false discovery rate (FDR) by defining thresholds of decoy hit frequencies at particular mass measurement accuracy (measured in parts per million from theoretical), XCorr and delta-XCorr (dCn) values. TMT reporter intensity was quantified for all three multiplexes together using an in-house TMT-quantification algorithm. Only proteins with at least one unique peptide were quantified and used for downstream analysis. Statistical analyses and processing of TMT comparisons were performed using Microsoft Excel and the R statistical programming language (http://www.R-project.org). Statistical significance for both longitudinal analyses and differential expression analyses was determined *via* two-tailed Student’s *t* tests; Benjamini-Hochberg correction for multiple hypothesis testing was applied to generate FDR-adjusted *p*-values (FDR < 1%). Gene Ontology (GO) analysis was performed using ShinyGO and STRING-DB.^20,21^ For a description of TMT bridge-normalization, see Figure S1F.

## 3 Results and Discussion

### 3.1 TMT-based Quantification of C_2_C_12_ Proteome Dynamics in Normal and Tamoxifen-Treated Cells

**Figure 1:**
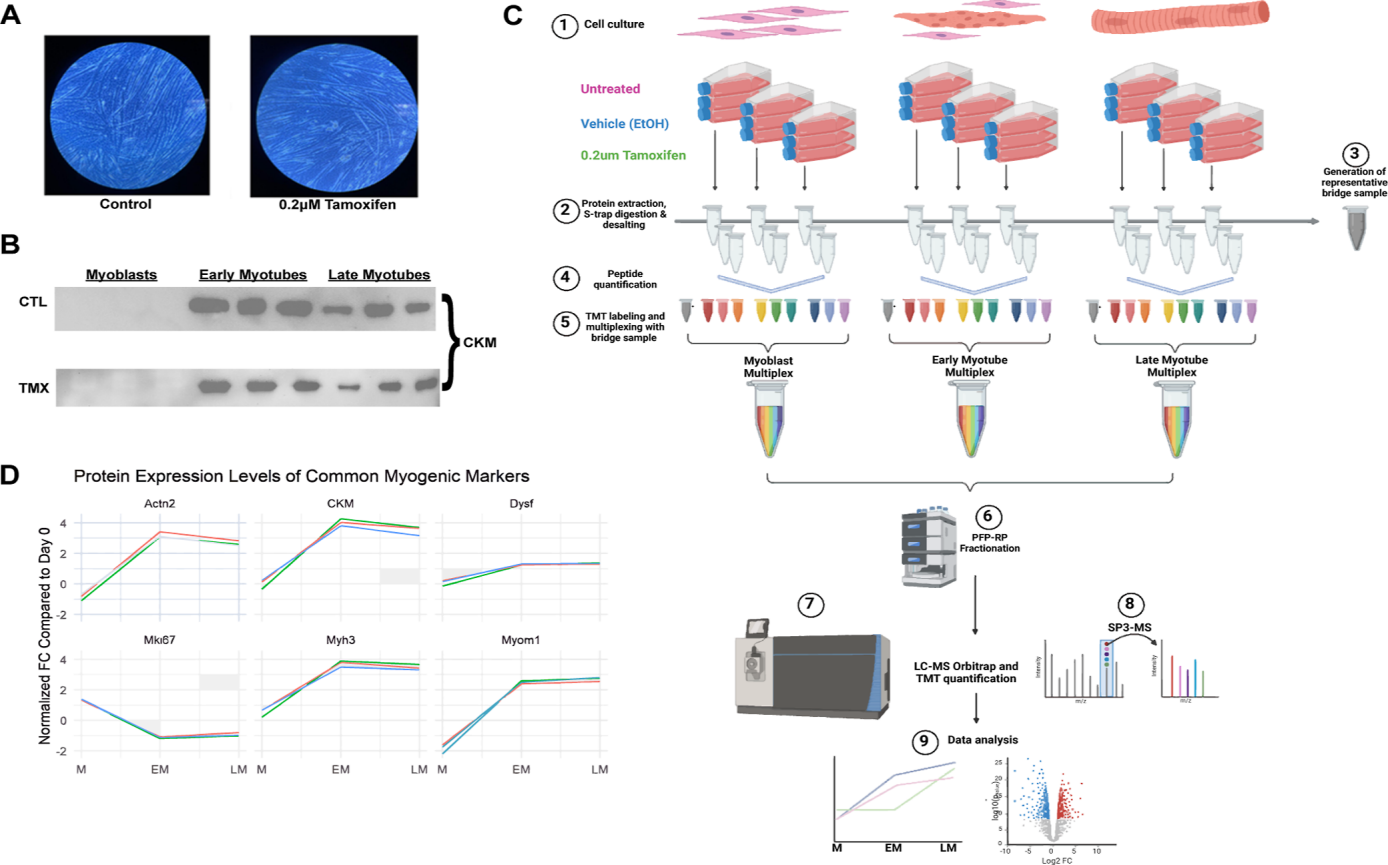
TMT-based quantification of C_2_C_12_ proteome dynamics in normal and TMX-treated cells. (A) Phenotypic comparison of untreated and TMX-treated myogenesis was performed to optimize a TMX concentration for proteomic experimentation; representative images of late myotubes grown with and without 0.2 μM TMX. (B) Myoblast differentiation was confirmed by WB against CKM. Protein was quantified by Lowry assay, and 20 μg of protein from *n* = 3 replicates per time point was loaded onto an SDS page for IB analysis. (C) Experimental overview of TMT workflow for proteome-wide analysis of C_2_C_12_ myoblast differentiation. (1) Cells were cultured in untreated, EtOH (vehicle), or EtOH + 0.2 μM TMX containing media (10% FBS). When cells reached confluency (day 0), proliferating cells were switched into differentiation media (5% HS) to induce differentiation into multinucleated myotubes. Cells were harvested from each treatment condition in triplicate at day 0 (myoblasts), day 5 (early myotubes), and day 9 (late myotubes). (2) Protein was extracted, quantified, and digested, and equal quantities of peptide from each sample were taken for TMT labeling. (3) Representative bridge sample was also generated using equal amounts of peptide from one sample from each treatment condition at each time point. (4) Samples were labeled using a TMT 10-plex kit to generate one multiplex per time point (myogenic stage), with *n* = 9 sample channels per multiplex plus *n* = 1 identical bridge sample as the 10th channel in each. (5,6) After confirming full labeling efficiency and equal mixing, each multiplex was separated first into 48 total fractions *via* PFP-RP-HPLC and then concatenated into 16 fractions for injection onto an Orbitrap Fusion mass spectrometer (7).^48^ (8) Peptides were sequenced, TMT reporter ions were analyzed using SPS-MS3, and peptides were identified and quantified using in-house software. (9) Relative abundance of each protein was first normalized to the average TMT channel intensity of all three multiplexes, then a bridge correction factor was calculated on a per protein basis from its bridge channel value to normalize day 5 and day 9 multiplexes to day 0. Analysis was performed longitudinally within each treatment condition to determine myogenically regulated proteins and within each multiplex to identify treatment-induced differential protein expression across the whole proteome. (D) Longitudinal analysis of common myogenic marker expression trajectories was performed to validate the TMT normalization scheme. Graphs show the standardized (median 0, std = 1) log_2_(average TMT intensity) at day 0 (M), followed by the standardized log_2_(FC) value of day 5 (EM) and day 9 (LM) compared to control; all six markers shown (ACTN2, CKM, DYSF, MKI67, MYH3, and MYOM1) had a significant (Benjamini-Hochberg corrected *p*-value < 0.05 by Student’s *t* test) log_2_(FC) relative to control at both day 5 and day 9 for each treatment condition.

A physiologically relevant concentration of TMX was determined based on the literature, as well as a dose–response assay testing four concentrations of TMX (0.1, 1, 10, and 100 μM, Figure S1A) for morphological effects.^51^ After establishing a concentration of TMX (0.2 μM Cayman Chemical Cat. #: 10540-29-1 in EtOH) that did not interfere with the rate of C_2_C_12_ proliferation and differentiation at a phenotypic level, we sought to conduct a quantitative analysis of the myogenic proteome of both untreated C_2_C_12_ cells and TMX-treated C_2_C_12_ cells (Figure 1C). As previous research has indicated a possible effect of EtOH (vehicle) alone on C_2_C_12_ development, we also chose to include a vehicle control-only condition separate from untreated controls, C_2_C_12_ myoblasts were thus cultured either untreated, in EtOH, or in EtOH + TMX (nine replicates each).^22^ When the myoblasts had proliferated to 80% confluency, media (10% FBS) was replaced with differentiation media (5% HS) containing the same treatment conditions. Cells were collected in triplicate at day 0 (myoblasts), 5 days (early myotubes), and 9 days (late myotubes) after myogenesis initiation. Myogenic progression was confirmed by phenotypic and western blot (WB) analyses of common myogenic markers (Figures 1C and S1B). Protein was harvested from lysed cells and digested; equal amounts (40 μg) of peptides were subsequently labeled with TMT reagents in triplicate. A representative bridge channel was also generated from these samples by combining equal amounts of peptide from each condition at each time point and dividing this combined sample into three (40 μg) bridge replicates. After confirming full labeling efficiency, labeled peptides were combined into three multiplexes: a myoblast multiplex, an early myotube multiplex, and a late myotube multiplex, each containing a 10th, identical bridge channel which allowed for normalization and comparison across all three multiplexes (Figure S1D,E). Each multiplex was then fractionated *via* RP-HPLC into 16 fractions, analyzed on an Orbitrap Fusion mass spectrometer, and quantified using in-house software. A detailed explanation of the experimental strategy is discussed in the Materials and Methods section and is visually summarized in Figure 1C.

Over 10,000 proteins were identified across all three multiplexes and used for downstream analysis (FDR < 1%) (Table S1). To validate the accuracy of our TMT tagging strategy and bridge normalization scheme, we performed a longitudinal analysis across all three multiplexes on a set of common myogenic markers (Figures S1E; 1D). The standardized Log2 Fold change (FC) of TMT intensities from myoblasts to early myotubes and myoblasts to late myotubes for these markers (Actn2, CKm, Dysf, Mki67, Myh3, and MyoM1) correlate to both WB data on the raw protein extracts and to existing literature on myogenic progression.^15^ We thus confirmed that our bridged-TMT model of myogenesis reflects biological conditions and could be used for further analysis of the myogenic proteome, as well as for treatment-based comparisons.

### 3.2 Temporal Proteome Profiling and Functional Characterization of Normal C_2_C_12_ Myogenesis

We next sought to establish a proteomic model of untreated C_2_C_12_ myogenesis using our TMT quantification strategy. From the >10,000 sequenced proteins, we identified a subset of 1,062 proteins that were DE throughout myogenesis in untreated C_2_C_12_ cells (Table S2). A protein was considered to be myogenically regulated if (a) the ratio of both EM to M and LM to M was significant (Benjamini-Hochberg adjusted *p*-value ≤ 0.05 by Student’s *t* test) and (b) the average, normalized log_2_(FC) of EM to M and/or LM to M had a magnitude of one or more (a fold change of ≥ |2|).

**Figure 2:**
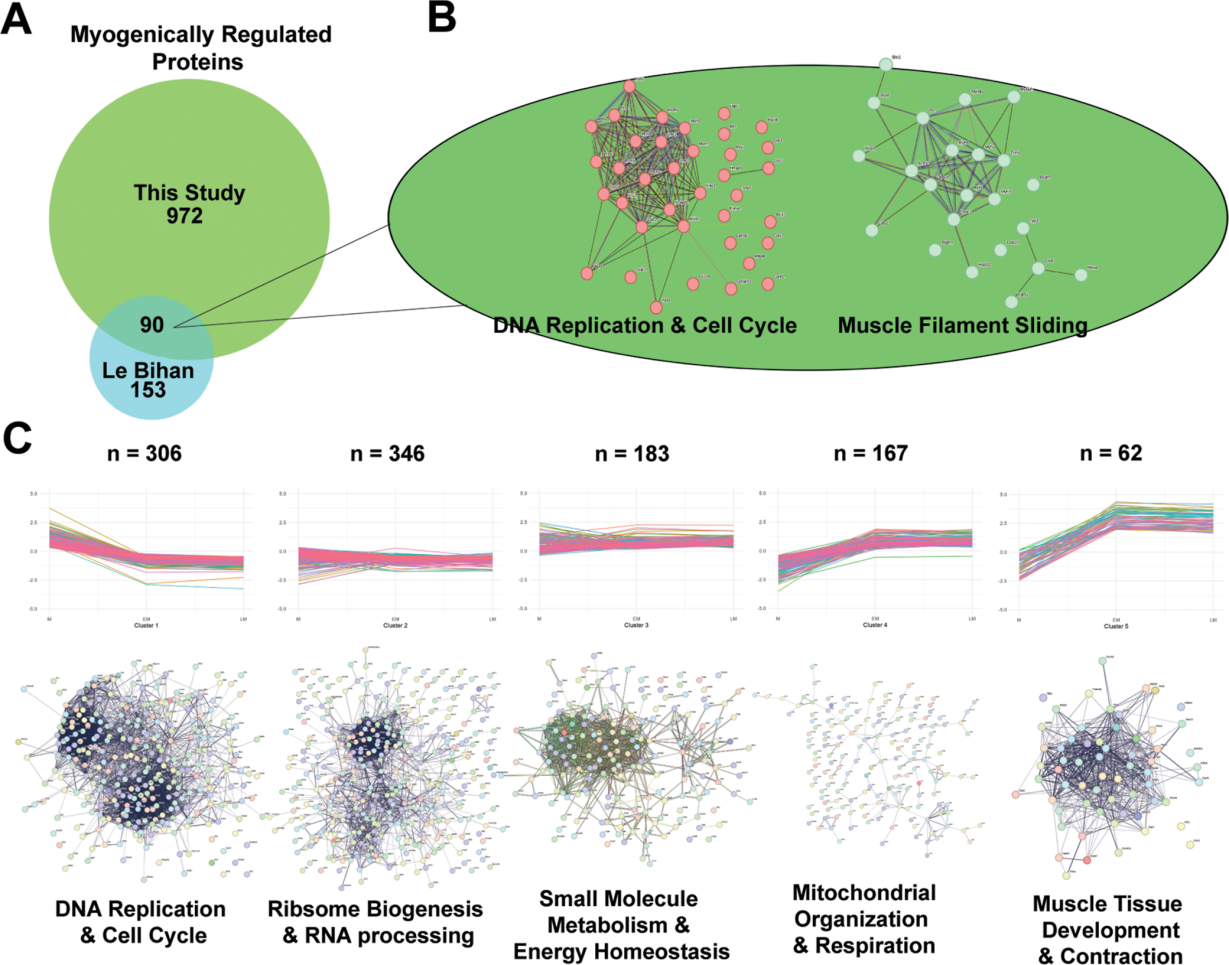
Temporal proteome profiling of normal C_2_C_12_ differentiation. Proteins with significant fold changes (Benjamini-Hochberg corrected *p*-value < 0.05 by Student’s *t* test) at both day 5 and day 9 relative to control, and which had a magnitude of change of 2 or more at either day 5, day 9, or both, were considered to be myogenically regulated (MR) in the untreated myogenesis analysis (*n* = 1,062). (A) Proteins identified as MR in this study were compared to the existing literature, and overlapping proteins were analyzed using STRING-db.^49^ (B) Trends in myogenic protein expression were assessed by subjecting the subset of MR proteins to k-means clustering (https://rdrr.io/r/stats/kmeans.html/), resulting in five functionally distinct protein expression clusters. Functional enrichment and network analysis was performed using [20,21] and summarized here; full networks and enrichment maps can be viewed in Figure S2B–D and in Tables S4 and S5.

While several proteomic data sets chronicling C_2_C_12_ myogenesis exist, the most comparable, quantitative analysis is a SILAC-based analysis of primary human myocyte differentiation in which 243 myogenically regulated proteins were identified, 90 of which overlapped to myogenically regulated mouse homolog proteins in our data set (Figure 2A; Table S3).^15^ STRING-DB analysis of these overlapping proteins revealed an enrichment for proteins involved in, predictably, muscle contraction and DNA replication/cell cycle control (Figure 2B).^21^ Our study and that of Le Bihan used different cell systems, quantification models, and instrumentation pipelines; furthermore, data-dependent acquisition mass spectrometry is semi-stochastic, and two identical sample runs on the same instrument can vary in peptide IDs by >40%.^15,47^ Nevertheless, our subset of myogenically regulated proteins overlaps with 37% of those identified in Le Bihan, suggesting not only reproducibility between the two approaches but also reflects the similarity of processes that underlie mouse and human myogenesis.^15^

To further characterize the protein expression patterns during myogenesis, we applied K-means clustering to the 1,062 myogenically regulated proteins to analyze longitudinal changes in expression over time. Based on the results of several iterations, as well as comparisons to existing literature, we find five clusters optimally encompass the longitudinal changes in our data set. GO and STRING analyses of these clusters revealed five distinct patterns of protein expression in the myogenic program (Figures 2C; S2A–F; Tables S2 and S4). The largest cluster (*n* = 306), cluster 1, decreased continuously through the process of differentiation and was significantly enriched for proteins involved in DNA replication and cell cycle control. As expected, the increase in M cell fusion to EM and the concomitant exit from the cell cycle to G_0_ readily accounts for the reduction in the level of proteins associated with DNA replication. Cluster 2 (*n* = 346) decreases in the transition from myoblasts to early myotubes but subsequently increases in the late myotube stage. The proteins included in cluster 2 primarily function in ribosome biogenesis and RNA processing. Prior to fusion, myoblasts in a rat cell line (L5/A10) express a non-muscle myosin II and a Ca^2+^-independent myosin light chain kinase, whereas post-fusion late myoblasts express an “adult” skeletal muscle type II myosin, as well as a Ca^2+^-dependent myosin light chain kinase.^31^ Fusion leads to a myofibrillogenic gene expression program that is accompanied by a significant increase in protein expression, possibly accounting for the observed increase in ribosome biogenesis and RNA processing proteins.^32^ In contrast to cluster 2, cluster 3 (*n* = 182) expression peaks at the EM stage and was enriched for proteins involved in small-molecule metabolism and energy homeostasis. As M transition to spontaneously contracting LM, the requirement for increased ATP synthesis and flux is demonstrated by the increase in citrate synthase as a marker for the increasing abundance of tricarboxylic acid cycle-related proteins.^33,34^ The two final clusters, cluster 4 (*n* = 166) and cluster 5 (*n* = 62), both increase in expression over time. Expression of proteins in cluster 4 increased gradually from the myoblast to late myotube stage and are primarily involved in mitochondrial organization and respiration, reflecting the increasing energy needs of maturing skeletal muscle cells. Similarly, the proteins in cluster 5—which quadruple in expression levels between the M and EM stages and remain elevated throughout the formation of late myotubes—are specifically involved in muscle contraction and skeletal muscle formation. Their rapid increase in abundance reflects the high level of organizational complexity necessary for the sarcomere structure and muscle contraction.^35^ Full network maps and corresponding enrichments can be found in Figure S2A–F and Tables S4 and S5.

Clusters 1, 3, and 5 demonstrated high levels of network interconnectivity by STRING analysis, while proteins in clusters 2 and 4 appear more loosely linked. Although there was significant enrichment for mitochondrial-related proteins, as well as for some muscle function-specific proteins in cluster 4, it was by far the most loosely networked of the five. Secondary K-means and MCL clustering of proteins within cluster 4 by STRING network interactions do not provide further insights into the functional nature of proteins within this cluster. However, based on trajectory, overall enrichment for mitochondrial organization suggests that the mitochondria are important for myogenesis progression and supports the idea of a metabolic shift from glycolysis to oxidative phosphorylation as development proceeds.^23^

### 3.3 Tamoxifen Induces Changes in Myogenic Adiponectin Signaling but Does Not Affect the Overall Program of Myogenic Protein Expression

**Figure 3:**
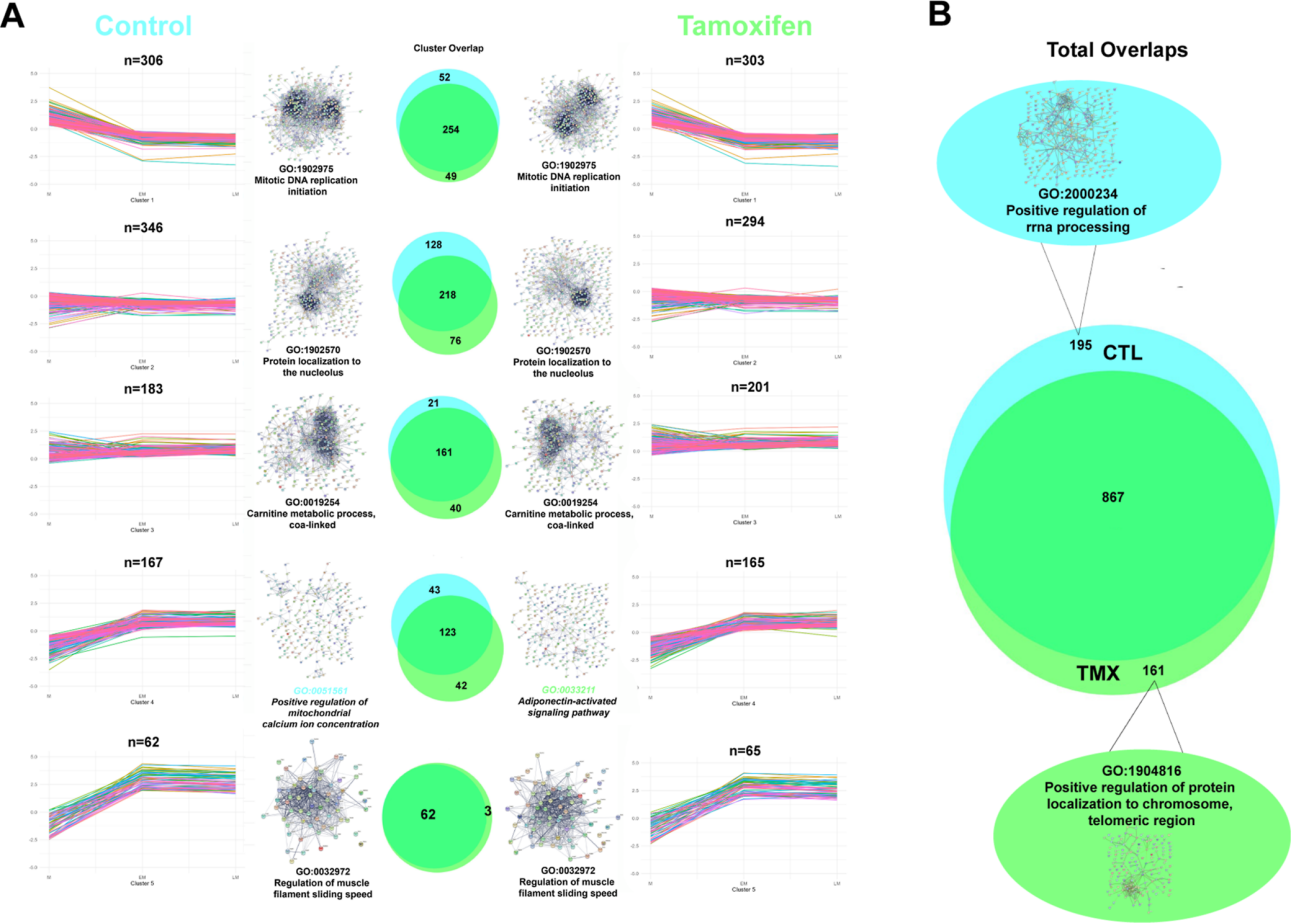
Effects of TMX on the myogenic program. (A) Identical clustering analysis and functional enrichment were performed on TMX-treated myogenesis. Proteins that were classified as MR only when EtOH (Vehicle) was present (*n* = 150) were excluded from TMX longitudinal analysis to correct for any effect of the vehicle/TMX interaction. A side-by-side comparison of untreated (control) MR protein clusters and TMX MR protein clusters is shown here. Network functional enrichments are summarized by the top GO/BP term from STRING-db analysis, and individual protein overlaps of each cluster are shown in the Venn diagrams.^49^ Full networks, functional enrichments, and cluster comparisons can be found in Table S6 and Figure S3. (B) MR proteins that were unique either to untreated myogenesis only or to TMX-treated myogenesis were analyzed en masse and assessed *via* functional enrichment; top GO/BP terms are shown; and complete enrichments can be found in Table S6.^49^

After full proteomic characterization of myogenesis in untreated cell lines, we applied the same analyses to the EtOH (vehicle) and TMX-treated resulting data sets. While the vast majority (*n* = 726) of myogenically regulated proteins did not differ across treatments, specific, identifiable subsets of proteins were DE in the EtOH-only and/or the EtOH + TMX-treated cells (Figure S3A; Tables S4, S5 and S6). Full comparison of untreated and vehicle control myogenesis can be found in Figure S3B–F. To remove the vehicle effect from downstream analysis of TMX myogenesis, we limited K-means clustering analysis to the subset of proteins that were myogenically regulated in all three conditions (*n* = 726), regulated in TMX myogenesis only (*n* = 161), or regulated in untreated and TMX-treated myogenesis only (*n* = 141) (Figure S3A). This resulted in removal of 150 out of 1,178 proteins from the TMX-treated data set that were myogenically regulated only in the presence of EtOH. The remaining 1,028 proteins were clustered using K-means analysis (Figure 3A). The five overall trends in expression were unchanged between untreated and TMX-treated myogenesis, and the component proteins exhibit significant overlap, although neither the number nor the identities of overlapping proteins between control and TMX-treated clusters were identical (with the exception of cluster 5). STRING analysis reveals similar networks operating in EtOH and TMX-treated cells; a comparison of top-most enriched GO-terms reveals that clusters 1, 2, 3, and 5 were functionally unaffected by TMX treatment. Interestingly, the proteins in TMX cluster 4, despite demonstrating the same patterns of expression as those in untreated cluster 4, are specifically enriched for adiponectin signaling. Both TMX and estrogen have previously been shown to alter adiponectin signaling in both androgenic and anabolic tissues; to the best of our knowledge, this is the first demonstration of this effect in a myogenesis model at the proteomic level.^24,25^

To determine if there were significant functional changes induced by TMX across all myogenically regulated proteins, we next performed STRING and GO enrichment on the subset of proteins that was unique to untreated myogenesis (*n* = 195) and TMX myogenesis (*n* = 161), respectively. Functional enrichment showed that the majority of proteins found only in the untreated data set were involved in rRNA processing, whereas the TMX effect was enriched for protein localization to telomeric DNA (Figure 3B). This may be due to DNA damage induced by TMX which is known to form adducts with DNA and have intercalative properties; more follow-up is necessary to confirm this effect in a C_2_C_12_ myogenesis model.^26^

### 3.4 Tamoxifen Treatment Alters Expression of Proteins Involved in Cholesterol, Steroid, and Lipid Storage at Different Stages of C_2_C_12_ Myogenesis

**Figure 4:**
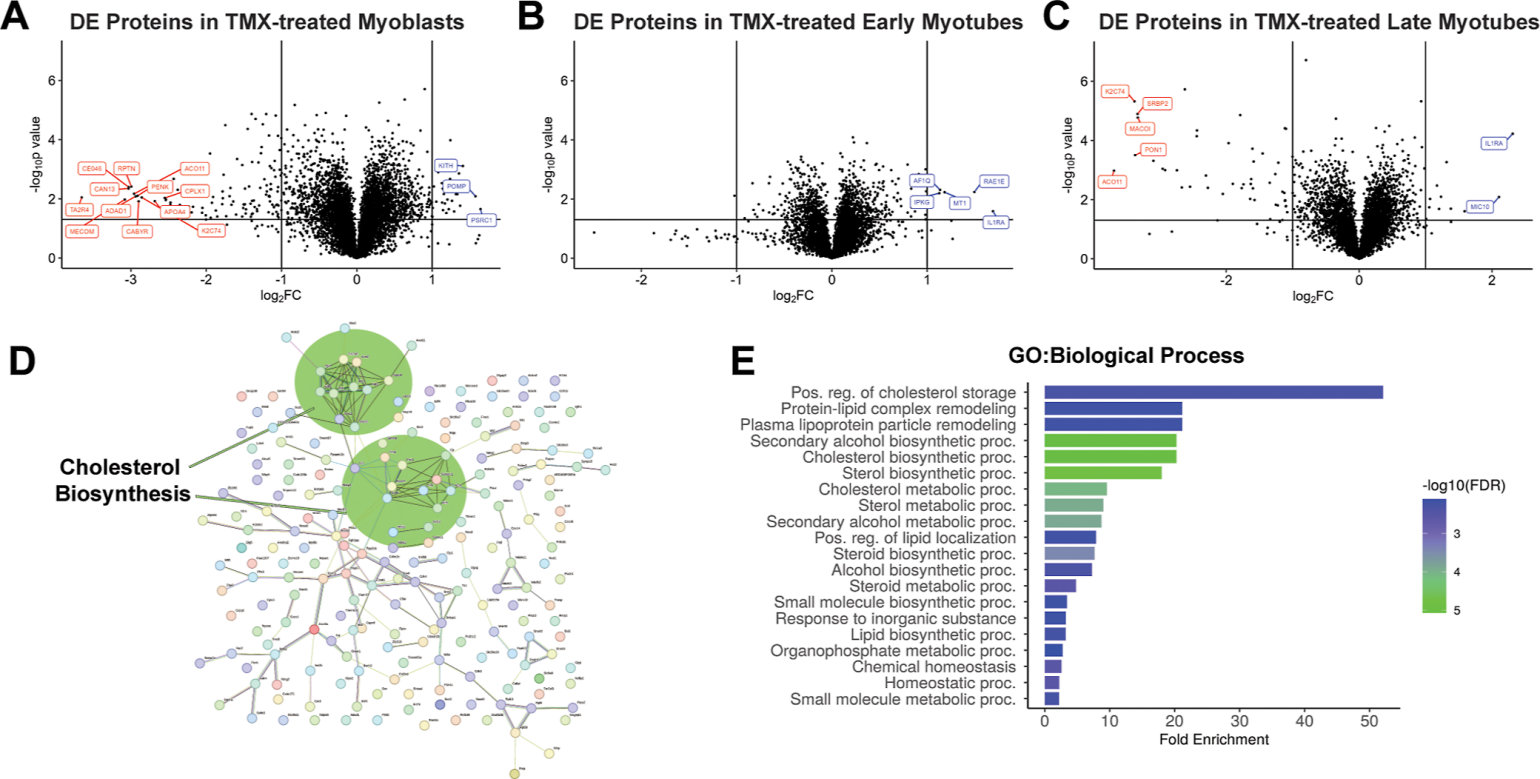
Effect of TMX on the total proteome of C_2_C_12_ cells at each stage of myogenesis. Differential expression of proteins was assessed by first normalizing vehicle and TMX + vehicle TMT values to the average per-protein untreated intensity values to remove vehicle/TMX interactions. Proteins were considered to be differentially expressed (DE) if log_2_(TMX-corrected/Vehicle-corrected) had a magnitude of 1 or more (two-fold change) and were significant (*p* < 0.05) by Student’s *t* test [-log_10_(BH-adjusted *p* value) ≥ 1.3]. (A) DE proteins in myoblasts; proteins with a FC in the top 10% in the positive or negative direction are labeled by protein name. (B) DE proteins in early myotubes; all DE proteins are labeled. (C) DE proteins in late myotubes; proteins with a FC in the top 10% in the positive or negative direction are labeled. (D) STRING-db network analysis and enrichment of all DE proteins. (E) ShinyGO GO/BP enrichment analysis of all DE proteins. Full protein lists and enrichments can be found in Table S7.

While analysis of myogenesis in the presence of TMX across all three TMT-multiplexes provided a longitudinal picture of TMX effects on myogenically regulated proteins, our experimental design also allows us to compare treatment conditions within each multiplex (myogenic stage) across the whole proteome. We first compared vehicle control to control and determined that EtOH alone had a significant effect on the proteome outside of the myogenic program (Figure S4A–H). To remove any potential interactions of EtOH and TMX from the analysis, we corrected the log_2_ value for TMT intensity of the vehicle control and the TMX + vehicle within each multiplex to the average log_2_ value from the untreated condition for each protein (Figure S4J). We then calculated the log_2_ (FC) of corrected-TMX TMT values to corrected-vehicle control values and performed Student’s *t* tests (Benjamini-Hochberg corrected *p*-value ≤ 0.05) on a per-protein level to identify DE proteins at each myogenic stage (M, EM, and LM). The majority of DE proteins were found in the M (*n* = 145) and LM stages (*n* = 63), whereas only five proteins were found to be DE at the EM stage (Figure 4A–C). Furthermore, the majority of DE proteins were downregulated relative to controls, suggesting that estrogen signaling may otherwise be responsible for maintaining expression of these proteins during normal myogenesis in these cells.

**Figure 5:**
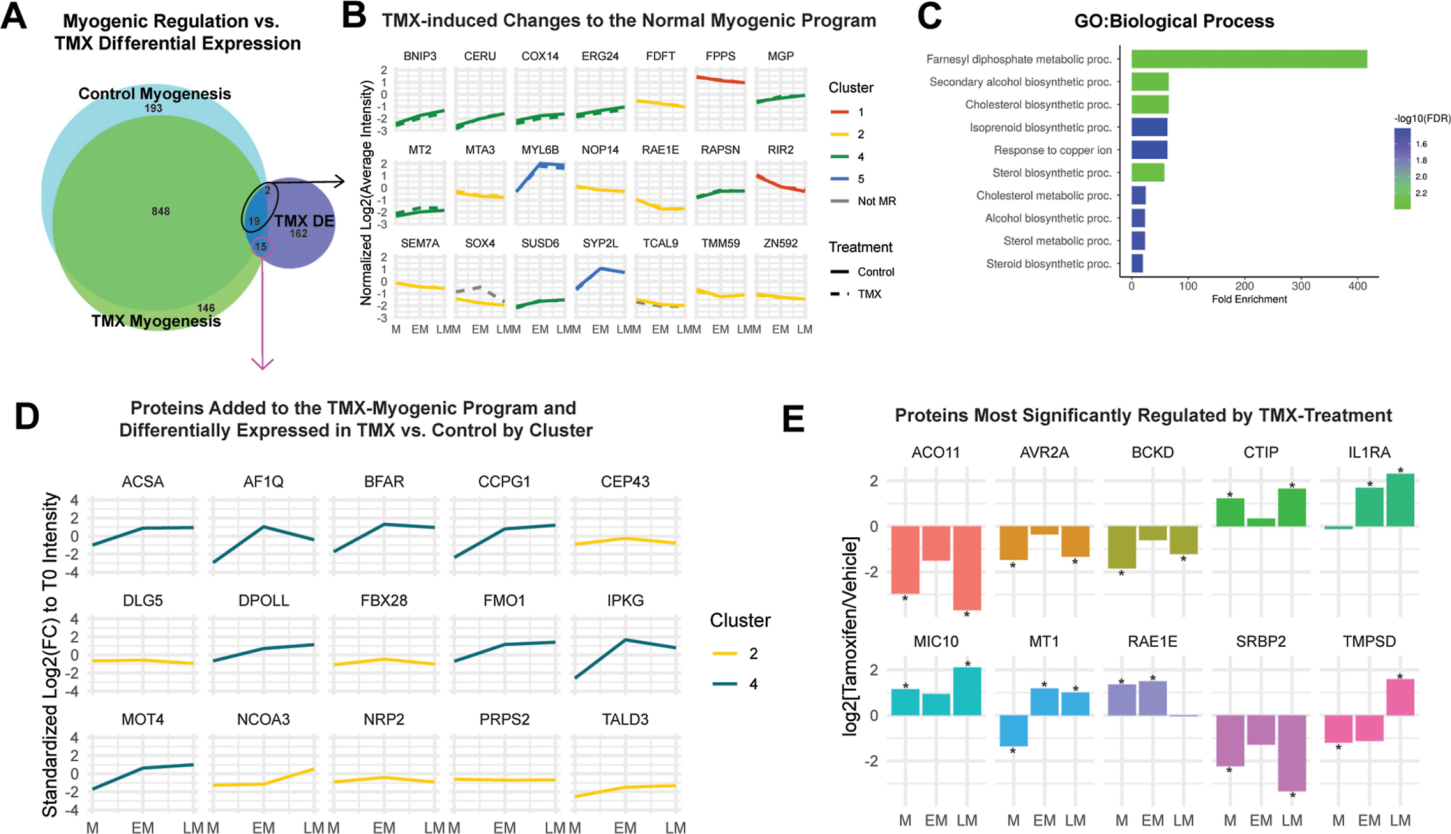
Identification of proteins most significantly regulated by TMX across developing C_2_C_12_ myocytes. (A) Overlaps of untreated MR proteins, TMX-treated MR proteins, and TMX DE proteins.^49^ (B) DE proteins that were also found to be MR in untreated myogenesis were assessed to determine if cluster assignment changed as a result of TMX-induced DE. Standardized (median = 0, std = 1) log_2_(average TMT intensity values) are shown for each treatment condition (note: average intensity values were scaled on a per treatment basis to show general expression trends, and trend lines are not representative of the actual DE ratio of TMX relative to controls). (C) GO/BP functional enrichment of proteins from (B). (D) Proteins that were added to the myogenic program as a result of TMX treatment were assessed to determine longitudinal cluster representation. Graphs show standardized log_2_(average M) values, followed by standardized log_2_(FC) of EM to M and LM to M ratios. Fold changes at D5 and D9 relative to control were significant (BH-adjusted *p* < 0.05) by Student’s *t* test for each protein. Eight out of 12 proteins (ACSA, AF1Q, BFAR, CCPG1, DPOLL, FMO1, IPKG, and MOT4) fell into cluster 4 of the TMX-longitudinal analysis, while the remaining six proteins (CEP43, DLG5 FBX28, NCOA3, PRPS2, and TALD3) were grouped into cluster 2 of TMX-longitudinal analysis. (E) Proteins were considered to be highly regulated by TMX treatment if they were DE (log_2_FC >|1| and BH-adjusted *p* < 0.05 by Student’s *t* test) at two or more time points relative to controls. 10 proteins (ACO11, AV2RA, BCKD, CTIP, IL1RA, MIC10, MT1, RAE1E, TMPSD, and SRBP2) were determined to be highly regulated by TMX, but only MT1 was significant at all three time points (* = *p*-value <0.05).

In total, we find 198 proteins that are expressed differentially at one or more myogenic stages as a result of TMX treatment (Table S5). While some of these proteins overlapped with the existing myogenic program (Figure 5A), most of the effects associated with TMX exposure are seen outside the myogenic program. Functional analysis of these 198 proteins revealed a specific enrichment for proteins involved in positive regulation of cholesterol, lipid, and sterol storage and metabolism (Figure 4D,E). When we analyzed downregulated and upregulated proteins separately, we found that specifically downregulated proteins were enriched for these pathways, suggesting a potential decrease in lipid-based energy storage and metabolism (Figures 4F; S5A,B). Recently, a complex interaction between lipid vesicles in C_2_C_12_ myoblasts and enhancement of myogenesis and myosin protein expression have been demonstrated.^36^ Interestingly, this effect appears to complement the differences found in longitudinal assessment of control versus TMX cluster 4, where TMX treatment resulted in a shift in enrichment from mitochondrial organization and regulation to proteins involved in adiponectin signaling (Figure 3A).

### 3.5 Identification of Proteins Most Significantly Regulated by Tamoxifen Treatment in Developing C_2_C_12_ Myocytes

By comparing the effects of TMX on the whole proteome to its effects on the subset of myogenically regulated proteins, we find that of the 198 proteins DE as a result of TMX treatment, 21 of them are also myogenically regulated in control myogenesis (Figure 5A). 19 of the 21 are myogenically regulated in both untreated and TMX-treated myogenesis, and the majority of these proteins are placed in untreated cluster 2 (ribosome biogenesis and rRNA processing) and untreated cluster 3 (small molecule metabolism and energy homeostasis) (Figure 5B). The other two proteins, transcription factor SOX-4 and transcription elongation factor A protein-like 9 (Tcal9), were no longer regulated as a function of myogenesis in the presence of TMX (Figure 5B). GO functional analysis of this 21-protein subset is enriched for metal ion homeostasis, as well as for cholesterol metabolism (Figure 5C).

Assessment of the subset of 15 DE proteins that are myogenically regulated in TMX-treated myogenesis but not in untreated myogenesis (*i.e.*, added to the myogenic program by TMX treatment) show no specific functional enrichment: all 15 proteins cluster into either cluster 2 or 4 of the TMX longitudinal analysis, with over half falling into cluster 4 (Figure 5D). This clustering is further supported by the results of our longitudinal comparison of TMX-treated to untreated myogenesis, where TMX cluster 4 is functionally distinct from untreated cluster 4 despite maintaining the same expression trend (Figure 2A). Likewise, clusters 2 and 4 were the most divergent between treatment conditions in terms of the protein composition (Figure 2A).

Finally, a subset of 10 proteins were DE in TMX-treated cells when compared to controls at two or more time points: acyl-coenzyme A thioesterase 11 (ACO11); activin receptor type-2A (AVR2A); branched-chain alpha-ketoacid dehydrogenase kinase (BCKD); DNA endonuclease RBBP8 (CTIP); interleukin-1 receptor antagonist protein (IL1RA); MICOS complex subunit Mic10 (MIC10); metallothionein-1 (MT1), retinoic acid early inducible protein 1-epsilon (RAE1E); sterol regulatory element-binding protein 2 (SRBP2); and transmembrane protease serine 13 (TMPSD) (Figure 5E). Of these, MT1, which regulates metal ion homeostasis in response to glucocorticoids, was the only protein DE at all three stages of myogenesis as a result of TMX treatment (Figure 5E). Elevated levels of MT1 have been correlated with TMX resistance, relapse, and increased patient mortality in breast cancer studies.^26^ Similarly, a study investigating the effect of MT1 and MT2 in skeletal muscle *in vivo* and *in vitro* demonstrated an increase in MT1 associated with rodent sarcopenia, cachexia, and has been associated with muscle atrophy, as well as negative regulation of hypertrophic pathways such as Akt/mTOR.^37^ While further confirmation is necessary, our data suggest that TMX-induced upregulation of MT1 protein expression levels may contribute mechanistically to the muscle weakness and atrophy-related symptoms reported by patients taking TMX.^27,40,41^

In addition to MT1, CTIP, IL1RA, and SRBP2 have also been previously shown to be regulated by TMX in other models.^26,28−30^ During C_2_C_12_ myogenesis, CTIP protein expression is upregulated in TMX-treated myoblasts and late myotubes compared to controls (Figure 5E). CTIP—a BRCA1-interacting protein—is transcriptionally downregulated in TMX-resistant, ER + MCF-7 breast cancer cells, and its expression positively correlates with ER-mediated disease-free survival in neoadjuvant breast cancer therapy; significantly, re-introduction of CTIP expression in TMX-resistant cells restores sensitivity to the inhibitory growth effects of TMX.^28^ Despite an increase in CTIP protein levels, TMX (at biologically relevant concentrations) did not inhibit growth of C_2_C_12_ in our hands, supporting the hypothesis that TMX mediation of ER signaling has differential effects in anabolic versus androgenic tissue. Similarly, IL1RA, a modulator of immune and inflammatory response which is upregulated as a result of TMX in early and late myotubes in our model, is downregulated in androgenic (human breast) tissue *in vivo* in response to TMX treatment, suggesting divergent inflammatory signaling in anabolic tissue versus androgenic tissue in response to TMXadd.^24,38^ Finally, the transcription factor SRBP2 (also known as SREBP-2), a primary mediator of sterol and cholesterol metabolism, is a direct target of SERM signaling in non-anabolic models.^39,46^ In breast cancer, lymphoblastic, and hepatocytic cells, TMX induces SRBP2 overexpression and target gene transcription in an ER-independent manner.^46^ In our skeletal muscle model, however, SRBP2 was downregulated by TMX treatment in myoblasts and late myotubes (Figure 5E; early myotube expression showed a non-significant downward trend). Although our proteomics data do not capture SRBP2 phospho-activation status or transcriptional activity, it is once again worth noting that the effect of TMX on SRBP2 expression in ER + anabolic cells appears to be the opposite of that seen in androgenic and other tissues. The negative regulation of SRBP2 protein expression seen here also suggests a potential metabolic shift in TMX-treated muscle cells away from lipid-based energy storage and utilization.

To the best of our knowledge, the remaining six proteins, ACO11, AVR2A, BCKD, MIC10, RAE1E, and TMSPD are novel effectors of TMX in skeletal muscle myogenesis. While these six proteins have no known interactions with one another, ACO11, AVR2A, BCKD, MIC10, and RAE1E are involved in lipid metabolism and regulation, mitochondrial metabolism, or both; these results further support the lipid and energy metabolism-specific effect of TMX on muscle cells seen in our longitudinal and differential protein expression analysis.^42−45^ TMPSD does fall under this metabolism-focused pattern; however, TMPSD expression is known to promote breast cancer progression and resistance to multiple chemotherapies.^52^ Regulation of TMPSD protein expression by TMX in C_2_C_12_ cells in this study is novel but nonetheless corroborates existing literature on the protein.^52−54^ A full list of the proteins regulated by TMX treatment at two or more stages of myogenesis can be found in Table S9.

## 4 Conclusions

The present study employs the model myogenesis system of murine C_2_C_12_ cells and TMT mass spectrometry, yielding the most in-depth TMT-based quantification of C_2_C_12_ myogenesis to date. The protocols developed in this paper can be easily extended to the study of other drug interactions with early skeletal muscle development. We validate the use of a bridged TMT normalization for longitudinal analyses across three TMT multiplexes and identify more than 10,000 proteins, of which >1000 were regulated in expression as a function of myogenesis. This regulated subset demonstrates high overlap with those found in similar studies of human primary culture myocytes. Using these protocols, we determine the effects of a SERM, TMX at a physiologically relevant concentration, on the proteome of developing myocytes across the three myogenic stages. Longitudinal analysis of the overall myogenic program in the presence of TMX closely resembles that seen in untreated cells; however, we find a TMX-specific enrichment for proteins involved in adiponectin and lipid-related signaling. Further comparisons across the entire proteome at each individual stage of myogenesis identified a separate subset of 198 proteins outside the normal C_2_C_12_ myogenic program that were DE in TMX-treated cells versus controls. The effect of TMX, particularly in myoblasts and late myotubes, yielded a significant downregulation of proteins involved in cholesterol, lipid, and sterol biosynthesis and storage. We also identify a subset of 10 highly regulated proteins (DE in the presence of TMX at two or more stages of myogenesis), six of which are novel effectors of TMX, and one of which, MT1, suggests a possible mechanistic route for the muscle weakness and atrophy-related side effects commonly reported by patients taking TMX. However, more targeted studies in animal models will be necessary to fully understand TMX’s effect on skeletal muscle growth and repair. Finally, we observed a separate and quantifiable effect of EtOH on C_2_C_12_ myogenesis that should be investigated further. Although we were able to remove this effect from our analyses, for this reason, we suggest caution when using EtOH as a solvent in future proteomic studies involving TMX in the culture of developing cells.

## Supporting Information Available

TMX growth assay, effect of media conditions on protein levels across myogenesis stages by SDS gels, WBs of known myogenic markers, TMT tagging scheme and normalization scheme, overlaps of all regulated proteins across treatment conditions and by cluster with corresponding GO enrichments, comparison of vehicle control (EtOH) to untreated and vehicle control + TMX and vehicle effect removal scheme, and quantification of all proteins that were DE at two or more time points (PDF)
Clusters by average log_2_ TMT intensity and full STRING-DB networks by cluster (ZIP)
Raw TMT values for filtered proteins and normalized quantification values (XLSX)
Regulated proteins (XLSX)
Mouse homolog of regulated human proteins from Le Bihan *et al.*(15) and overlapping regulated proteins from Le Bihan with this study (XLSX)
Proteins by myogenic regulatory cluster (XLSX)
STRING-DB enrichments of untreated myogenic clusters (XLSX)
STRING-DB enrichments of vehicle control only, TMX-only, and control and TMX only MR enrichments (XLSX)
Proteins regulated by TMX across myogenesis and overlaps with DE proteins, detailed information on nine most regulated proteins, eggNOG IDs, and relevant citations (XLSX)
DE proteins and normalized TMT values (XLSX)

## Notes

The mass spectrometry proteomics data have been deposited to the MassIVE Archive (https://massive.ucsd.edu/ProteoSAFe/dataset.jsp?task=2943a28a82a2484bba0e58a1889758b7) *via* the MassIVE partner repository with the data set identifier PXD042803 with username MSV000092126_reviewer and password p119.

## Abbreviations

ACO11: acyl-coenzyme A thioesterase 11
AVR2A: activin receptor type-2A
BCKD: branched-chain alpha-ketoacid dehydrogenase kinase
BH: Benjamini-Hochberg
CTIP: DNA endonuclease RBBP8
CTRL: control
DDA: data-dependent acquisition
DE: differentially expressed
DMEM: Dulbecco’s Modified Eagle’s Medium
DMSO: dimethyl sulfoxide
EPPS: 3-[4-(2-hydroxyethyl)piperazin-1-yl]propane-1-sulfonic acid
ER: estrogen receptor
ERa: estrogen receptor alpha
ERb: estrogen receptor beta
EM: early myotube
EtOH: ethanol
FC: fold change
GO: gene ontology
GPER: G protein-coupled estrogen receptor
HS: horse serum
H_2_O: dihydrogen oxide
IL1RA: interleukin-1 receptor antagonist protein
LM: late myotube
M: myoblast
MIC10: MICOS complex subunit Mic10
MT1: metallothionein-1
NS: not significant
PFP: pentrafluorophosphate
RAE1E: retinoic acid early inducible protein 1-epsilon
RO: reverse osmosis
SERM: selective estrogen receptor modulator
SRBP2: sterol regulatory element-binding protein 2
TBS-T: tris-buffered saline and tween-20
TEAB: triethylammonium bicarbonate
TMPSD: transmembrane protease serine 13
TMX: tamoxifen
TMT: tandem mass-tag

## References

[ref1] Conboy I. M.; Rando T. A. The Regulation of Notch Signaling Controls Satellite Cell Activation and Cell Fate Determination in Postnatal Myogenesis. Dev. Cell 2002, 3, 397–409. 10.1016/S1534-5807(02)00254-X. 12361602

[ref2] Constantin B.; Cognard C.; Raymond G. Myoblast fusion requires cytosolic calcium elevation but not activation of voltage-dependent calcium channels. Cell Calcium 1996, 19, 365–374. 10.1016/s0143-4160(96)90109-8. 8793176

[ref3] Frontera W. R.; Ochala J. Skeletal Muscle: A Brief Review of Structure and Function. Calcif. Tissue Int. 2015, 96, 183–195. 10.1007/s00223-014-9915-y. 25294644

[ref4] Altuwaijri S.; Lee D. K.; Chuang K. H.; Ting H. J.; Yang Z.; Xu Q.; Tsai M. Y.; Yeh S.; Hanchett L. A.; Chang H. C.; Chang C. Androgen receptor regulates expression of skeletal muscle-specific proteins and muscle cell types. Endocrine 2004, 25, 27–32. 10.1385/endo:25:1:27. 15545703

[ref5] Rossetti M. L.; Steiner J. L.; Gordon B. S. Androgen-mediated regulation of skeletal muscle protein balance. Mol. Cell. Endocrinol. 2017, 447, 35–44. 10.1016/j.mce.2017.02.031. 28237723 PMC5407187

[ref6] Siegel R. L.; Miller K. D.; Fuchs H. E.; Jemal A. Cancer Statistics 2022. CA A Cancer J. Clin. 2022, 72, 7–33. 10.3322/caac.21708. 35020204

[ref7] Kohler B. A.; Sherman R. L.; Howlader N.; Jemal A.; Ryerson A. B.; Henry K. A.; Boscoe F. P.; Cronin K. A.; Lake A.; Noone A. M.; et al. Annual Report to the Nation on the Status of Cancer, 1975–2011, featuring incidence of breast cancer subtypes by race/ethnicity, poverty, and state. J. Natl. Cancer Inst. 2015, 107, djv048 10.1093/jnci/djv048. 25825511 PMC4603551

[ref8] Goodsell D. S. The Molecular Perspective: Tamoxifen and the Estrogen Receptor. Stem Cell. 2002, 20, 267–268. 10.1634/stemcells.20-3-267. 12004085

[ref9] Oh T. S.; Choi J. W.; Choi D. K.; Mukherjee R.; Liu H.; Yun J. W. Gender Dimorphism in Skeletal Muscle Proteome Between Lean and Diet-induced Obese Rats. Cell. Physiol. Biochem. 2011, 28, 981–996. 10.1159/000335811. 22178949

[ref10] Seko D.; Fujita R.; Kitajima Y.; Nakamura K.; Imai Y.; Ono Y. Estrogen receptor β controls muscle growth and regeneration in young female mice. Stem Cell Rep. 2020, 15, 577–586. 10.1016/j.stemcr.2020.07.017. PMC7486216 32822588

[ref11] Ikeda K.; Ito A.; Imada R.; Sato M.; Kawabe Y.; Kamihira M. In vitro drug testing based on contractile activity of C2C12 cells in an epigenetic drug model. Sci. Rep. 2017, 7, 44570 10.1038/srep44570. 28300163 PMC5353687

[ref12] Kislinger T.; Gramolini A. O.; Pan Y.; Rahman K.; MacLennan D. H.; Emili A. Proteome dynamics during C2C12 myoblast differentiation. Mol. Cell. Proteomics 2005, 4, 887–901. 10.1074/mcp.m400182-mcp200. 15824125

[ref13] Casadei L.; Vallorani L.; Gioacchini A. M.; Guescini M.; Burattini S.; D’Emilio A.; Biagiotti L.; Falcieri E.; Stocchi V. Proteomics-based investigation in C2C12 myoblast differentiation. Eur. J. Histochem. 2009, 53, e31 10.4081/ejh.2009.261. 22073363 PMC3167332

[ref14] Hutchinson-Bunch C.; Sanford J. A.; Hansen J. R.; Gritsenko M. A.; Rodland K. D.; Piehowski P. D.; Qian W. J.; Adkins J. N. Assessment of TMT Labeling Efficiency in Large-Scale Quantitative Proteomics: The Critical Effect of Sample pH. ACS Omega 2021, 6, 12660–12666. 10.1021/acsomega.1c00776. 34056417 PMC8154127

[ref15] Le Bihan M.-C.; Barrio-Hernandez I.; Mortensen T. P.; Henningsen J.; Jensen S. S.; Bigot A.; Blagoev B.; Butler-Browne G.; Kratchmarova I. Cellular Proteome Dynamics during Differentiation of Human Primary Myoblasts. J. Proteome Res. 2015, 14, 3348–3361. 10.1021/acs.jproteome.5b00397. 26074025

[ref16] Lowry O. H.; Rosebrough N.; Farr A. L.; Randall R. J. Protein measurement with the Folin phenol reagent. J. Biol. Chem. 1951, 193, 265–275. 10.1016/s0021-9258(19)52451-6. 14907713

[ref17] Laemmli U. K. Cleavage of structural proteins during the assembly of the head of bacteriophage T4. Nature 1970, 227, 680–685. 10.1038/227680a0. 5432063

[ref18] Towbin H.; Staehelin T.; Gordon J. Electrophoretic transfer of proteins from polyacrylamide gels to nitrocellulose sheets: procedure and some applications. Proc. Natl. Acad. Sci. U.S.A. 1979, 76, 4350–4354. 10.1073/pnas.76.9.4350. 388439 PMC411572

[ref19] Grassetti A. V.; Hards R.; Gerber S. A. Offline pentafluorophenyl (PFP)-RP prefractionation as an alternative to high-pH RP for comprehensive LC-MS/MS proteomics and phosphoproteomics. Anal. Bioanal. Chem. 2017, 409, 4615–4625. 10.1007/s00216-017-0407-6. 28555341 PMC5704945

[ref20] Ge S.; Jung D.; Yao R. ShinyGO: a graphical gene-set enrichment tool for animals and plants. Bioinformatics 2020, 36, 2628–2629. 10.1093/bioinformatics/btz931. 31882993 PMC7178415

[ref21] Szklarczyk D.; Gable A. L.; Nastou K. C.; Lyon D.; Kirsch R.; Pyysalo S.; Doncheva N. T.; Legeay M.; Fang T.; Bork P.; Jensen L. J.; von Mering C. The STRING database in 2021: customizable protein–protein networks, and functional characterization of user-uploaded gene/measurement sets. Nucleic Acids Res. 2021, 49, D605–D612. 10.1093/nar/gkaa1074. 33237311 PMC7779004

[ref22] Kielbasa O.; Brough C. Ethanol-induced inhibition of C2C12 muscle cell differentiation. J. Penn. Acad. Sci. 2017, 91, 11–21. 10.5325/jpennacadscie.91.1.0011.

[ref23] Wagatsuma A.; Sakuma K. Mitochondria as a Potential Regulator of Myogenesis. Sci. World J. 2013, 2013, 593267 10.1155/2013/593267. PMC3574753 23431256

[ref24] Morad V.; Abrahamsson A.; Dabrosin C. Estradiol affects extracellular leptin: adiponectin ratio in human breast tissue in vivo. J. Clin. Endocrinol. Metab. 2014, 99, 3460–3467. 10.1210/jc.2014-1129. 24796929

[ref25] Huang W. Y.; Chen D. R.; Kor C. T.; Chen T. Y.; Lin P. T.; Tseng J. T. C.; Wu H. M. Relationships between Follicle-Stimulating Hormone and Adiponectin in Postmenopausal Women. Metabolites 2020, 10, 420 10.3390/METABO10100420. 33086618 PMC7603381

[ref26] Surowiak P.; Materna V.; Kaplenko I.; Spaczyński M.; Dietel M.; Lage H.; Zabel M. Augmented expression of metallothionein and glutathione S-transferase pi as unfavourable prognostic factors in cisplatin-treated ovarian cancer patients. Virchows Arch. 2005, 447, 626–633. 10.1007/s00428-005-1228-0. 15968547

[ref27] Summermatter S.; Bouzan A.; Pierrel E.; Melly S.; Stauffer D.; Gutzwiller S.; Nolin E.; Dornelas C.; Fryer C.; Leighton-Davies J.; Glass D. J.; Fournier B. Blockade of Metallothioneins 1 and 2 Increases Skeletal Muscle Mass and Strength. Mol. Cell. Biol. 2017, 37, e00305–e00316. 10.1128/MCB.00305-16. 27956698 PMC5311239

[ref28] Wu M.; Soler D. R.; Abba M. C.; Nunez M. I.; Baer R.; Hatzis C.; Llombart-Cussac A.; Llombart-Bosch A.; Aldaz C. M. CtIP silencing as a novel mechanism of tamoxifen resistance in breast cancer. Mol. Cancer Res. 2007, 5, 1285–1295. 10.1158/1541-7786.MCR-07-0126. 18171986

[ref29] Chatterjee S.; Bhat V.; Berdnikov A.; Liu J.; Zhang G.; Buchel E.; Safneck J.; Marshall A. J.; Murphy L. C.; Postovit L. M.; Raouf A. Paracrine Crosstalk between Fibroblasts and ER+ Breast Cancer Cells Creates an IL1β-Enriched Niche that Promotes Tumor Growth. IScience 2019, 19, 388–401. 10.1016/J.ISCI.2019.07.034. 31419632 PMC6706609

[ref30] Vidal H.; Mondesert G.; Galiègue S.; Carrière D.; Dupuy P.-H.; Carayon P.; Combes T.; Bribes E.; Simony-Lafontaine J.; Kramar A.; Loison G.; Casellas P. Identification and Pharmacological Characterization of SRBP-2: A Novel SR31747A-binding Protein. Cancer Research, American Association for Cancer Research (AACR): 2003; Vol. 63. 12941800

[ref31] Scordilis S. P.; Uhlendorf B. W.; Scarpa S.; Cantoni G. L.; Miles J. M.; Adelstein R. S. Changes in myosin and myosin light chain kinase during myogenesis. Biochemistry 1981, 20, 3511–3516. 10.1021/bi00515a032. 6894861

[ref32] Abdelmoez A. M.; Sardón Puig L.; Smith J. A. B.; Gabriel B. M.; Savikj M.; Dollet L.; Chibalin A. V.; Krook A.; Zierath J. R.; Pillon N. J. Comparative profiling of skeletal muscle models reveals heterogeneity of transcriptome and metabolism. Am. J. Physiol. Cell Physiol. 2020, 318, C615–C626. 10.1152/ajpcell.00540.2019. 31825657 PMC7099524

[ref33] Vigelsø A.; Andersen N. B.; Dela F. The relationship between skeletal muscle mitochondrial citrate synthase activity and whole body oxygen uptake adaptations in response to exercise training. Int. J. Physiol., Pathophysiol. Pharmacol. 2014, 6, 84–101. 25057335 PMC4106645

[ref34] Blomstrand E.; Rådegran G.; Saltin B. Maximum rate of oxygen uptake by human skeletal muscle in relation to maximal activities of enzymes in the Krebs cycle. J. Physiol. 1997, 501, 455–460. 10.1111/j.1469-7793.1997.455bn.x. 9192316 PMC1159492

[ref35] Briggs R. T.; Scordilis S. P.; Powell J. A. Myofibrillogenesis in rodent skeletal muscle in vitro: two pathways involving thick filament aggregates. Tissue Cell 1995, 27, 91–104. 10.1016/s0040-8166(95)80014-x. 7740537

[ref36] Tan Y.; Jin Y.; Zhao P.; Wu J.; Ren Z. Lipid droplets contribute myogenic differentiation in C2C12 by promoting the remodeling of the acstin-filament. Cell Death Dis. 2021, 12, 1102 10.1038/s41419-021-04273-8. 34815388 PMC8611090

[ref37] Makharashvili N.; Paull T. T. CtIP: A DNA damage response protein at the intersection of DNA metabolism. DNA Repair 2015, 32, 75–81. 10.1016/j.dnarep.2015.04.016. 25957490

[ref38] Arend W. P.; Malyak M.; Guthridge C. J.; Gabay C. Interleukin-1 receptor antagonist: role in biology. Annu. Rev. Immunol. 1998, 16, 27–55. 10.1146/annurev.immunol.16.1.27. 9597123

[ref39] Mukherjee M.; Basu Ball W.; Das P. K. Das, Leishmania donovani activates SREBP2 to modulate macrophage membrane cholesterol and mitochondrial oxidants for establishment of infection. Int. J. Biochem. Cell Biol. 2014, 55, 196–208. 10.1016/j.biocel.2014.08.019. 25218172

[ref40] Khan Q. J.; O’Dea A. P.; Sharma P. Musculoskeletal adverse events associated with adjuvant aromatase inhibitors. J. Oncol. 2010, 2010, 654348 10.1155/2010/654348. 20871846 PMC2943085

[ref41] Mourits M. J.; Bö Ckermann I.; de Vries E. G.; van der Zee A. G.; ten Hoor K. A.; van der Graaf W. T.; Sluiter W. J.; Willemse P. H. Tamoxifen effects on subjective and psychosexual well-being, in a randomised breast cancer study comparing high-dose and standard-dose chemotherapy. Br. J. Cancer 2002, 86, 1546–1550. 10.1038/sj/bjc/6600294. 12085202 PMC2746594

[ref42] Rampelt H.; Bohnert M.; Zerbes R. M.; Horvath S. E.; Warscheid B.; Pfanner N.; van der Laan M. Mic10, a Core Subunit of the Mitochondrial Contact Site and Cristae Organizing System, Interacts with the Dimeric F1Fo-ATP Synthase. J. Mol. Biol. 2017, 429, 1162–1170. 10.1016/J.JMB.2017.03.006. 28315355

[ref43] Adams S. H.; Chui C.; Schilbach S. L.; Yu X. X.; Goddard A. D.; Grimaldi J. C.; Lee J.; Dowd P.; Colman S.; Lewin D. A. BFIT, a unique acyl-CoA thioesterase induced in thermogenic brown adipose tissue: cloning, organization of the human gene and assessment of a potential link to obesity. Biochem. J. 2001, 360, 135–142. 10.1042/0264-6021:3600135. 11696000 PMC1222210

[ref44] Trembath A. P.; Sharma N.; Markiewicz M. A. NKG2D signaling within the pancreas enhances CD8+ central memory T cell formation and decreases non-obese diabetic (NOD) diabetes development. J. Immunol. 2018, 200, 163.22 10.4049/jimmunol.200.supp.163.22.29187586

[ref45] Dani C. Activins in adipogenesis and obesity. Int. J. Obes. 2013, 37, 163–166. 10.1038/ijo.2012.28. 22370854

[ref46] Fernández-Suárez M. E.; Daimiel L.; Villa-Turégano G.; Pavón M. V.; Busto R.; Escolà-Gil J. C.; Platt F. M.; Lasunción M. A.; Martínez-Botas J.; GómezCoronado D. Selective estrogen receptor modulators (SERMs) affect cholesterol homeostasis through the master regulators SREBP and LXR. Biomed. Pharmacother. 2021, 141, 111871 10.1016/J.BIOPHA.2021.111871. 34225017

[ref47] Tabb D. L.; Vega-Montoto L.; Rudnick P. A.; Variyath A. M.; Ham A. J. L.; Bunk D. M.; Kilpatrick L. E.; Billheimer D. D.; Blackman R. K.; Cardasis H. L.; Carr S. A.; Clauser K. R.; Jaffe J. D.; Kowalski K. A.; Neubert T. A.; Regnier F. E.; Schilling B.; Tegeler T. J.; Wang M.; Wang P.; et al. Repeatability and reproducibility in proteomic identifications by liquid chromatography-tandem mass spectrometry. J. Proteome Res. 2010, 9, 761–776. 10.1021/PR9006365/SUPPL_FILE/PR9006365_SI_008.XLS. 19921851 PMC2818771

[ref48] ″http://Biorender.com″>Biorender.com.

[ref49] Hulsen T.; de Vlieg J.; Alkema W. BioVenn - a web application for the comparison and visualization of biological lists using area-proportional Venn diagrams. BMC Genom. 2008, 9, 488 10.1186/1471-2164-9-488. PMC2584113 18925949

[ref50] Vogel C.; Marcotte E. M. Insights into the regulation of protein abundance from proteomic and transcriptomic analyses. Nat. Rev. Genet. 2012, 13, 227–232. 10.1038/nrg3185. 22411467 PMC3654667

[ref51] Gjerde J.; Gandini S.; Guerrieri-Gonzaga A.; Haugan Moi L. L.; Aristarco V.; Mellgren G.; Decensi A.; Lien E. A. Tissue distribution of 4-hydroxy-N-desmethyltamoxifen and tamoxifen-N-oxide. Breast Cancer Res. Treat. 2012, 134, 693–700. 10.1007/s10549-012-2074-9. 22562123 PMC3401496

[ref52] Murray A. S.; Hyland T. E.; Sala-Hamrick K. E.; Mackinder J. R.; Martin C. E.; Tanabe L. M.; Varela F. A.; List K. The cell-surface anchored serine protease TMPRSS13 promotes breast cancer progression and resistance to chemotherapy. Oncogene 2020, 39, 6421–6436. 10.1038/s41388-020-01436-3. 32868877 PMC8143875

[ref53] Hsieh E. J.; Hoopmann M. R.; MacLean B.; MacCoss M. J. Comparison of database search strategies for high precursor mass accuracy MS/MS data. J. Proteome Res. 2010, 9, 1138–1143. 10.1021/pr900816a. 19938873 PMC2818881

[ref54] Varela F. A.; Foust V. L.; Hyland T. E.; Sala-Hamrick K. E.; Mackinder J. R.; Martin C. E.; Murray A. S.; Todi S. V.; List K. TMPRSS13 promotes cell survival, invasion, and resistance to drug-induced apoptosis in colorectal cancer. Sci. Rep. 2020, 10, 13896 10.1038/s41598-020-70636-4. 32807808 PMC7431588

