# Supplementary material for: Effect of Tamoxifen on Proteome Expression during In Vitro Myogenesis in Murine Skeletal Muscle C2C12 Cells
Source: J Proteome Res. 2023 Aug 8;22(9):3040–53. doi: 10.1021/acs.jproteome.3c00340 (PMC10476267; doi:10.1021/acs.jproteome.3c00340)
Supplement: Supplementary file 1 — pr3c00340_si_001.pdf [file pr3c00340_si_001.pdf]

The Effect of Tamoxifen on Proteome Expression During *in vitro* Myogenesis in Murine Skeletal Muscle C_2C_{12} Cells: Supporting Information

Emily A. Morris[1a], Ahlenne Abreu[2a] and Stylianos P Scordilis[3*]

[1] Department of Microbiology and Immunology, Geisel School of Medicine at Dartmouth, Borwell Building 644E, Lebanon, NH 03756 United States of America email: em.morris.gr@dartmouth.edu

[2] Department of Cancer Biology, Perelman School of Medicine, 421 Curie Blvd. Room 612 BRB II/III University of Pennsylvania Medical School Philadelphia, PA 19104 United States of America ahlenne@upenn.edu

[3]Department of Biological Sciences, Smith College, Ford Hall 202 B, Northampton, MA 01063 United States of America sscordil@smith.edu

[a]These co-authors contributed equally to this work.

*Corresponding Author: Stylianos P Scordilis, PhD

Ford Hall 202 B, 100 Green Street, Smith College, Northampton MA 01063 United States of America - Email: sscordil@smith.edu

Supplementary Figures

Figure S1: Tamoxifen growth assay, effect of media conditions on protein levels across myogenesis stages by SDS gels, WBs of known myogenic markers, TMT tagging scheme and normalization scheme.
Figure S2: Clusters by average log2 TMT intensity; full STRING-DB networks by cluster are in separate documents (S2B-S2F .svg files included with supplemental data).
Figure S3: Overlaps of all regulated proteins across treatment conditions and by cluster with corresponding GO enrichments.
Figure S4: Comparison of vehicle control (EtOH) to untreated & vehicle control + tamoxifen and vehicle effect removal scheme.
Figure S5: Quantification of all proteins that were DE at 2 or more time points.

Supplementary Tables

Table S1: Raw TMT values for filtered proteins and normalized quantification values .
Table S2: Complete list of regulated proteins.
Table S3: Mouse homolog of regulated human proteins from le Bihan *et al.,* 2015 and overlapping regulated proteins from le Bihan with this study.
Table S4: Proteins by myogenic regulatory cluster.
Table S5: STRING-DB enrichments of untreated myogenic clusters.
Table S6: STRING-DB enrichments of vehicle control only, TMX-only, and control & TMX only MR enrichments.
Table S7: Full list of differentially expressed proteins and normalized TMT values.
Table S8: Full list of proteins regulated by TMX across myogenesis and overlaps with differentially expressed proteins.
Table S9: Detailed information on 9 most regulated proteins, eggNOG IDs, and relevant citations.

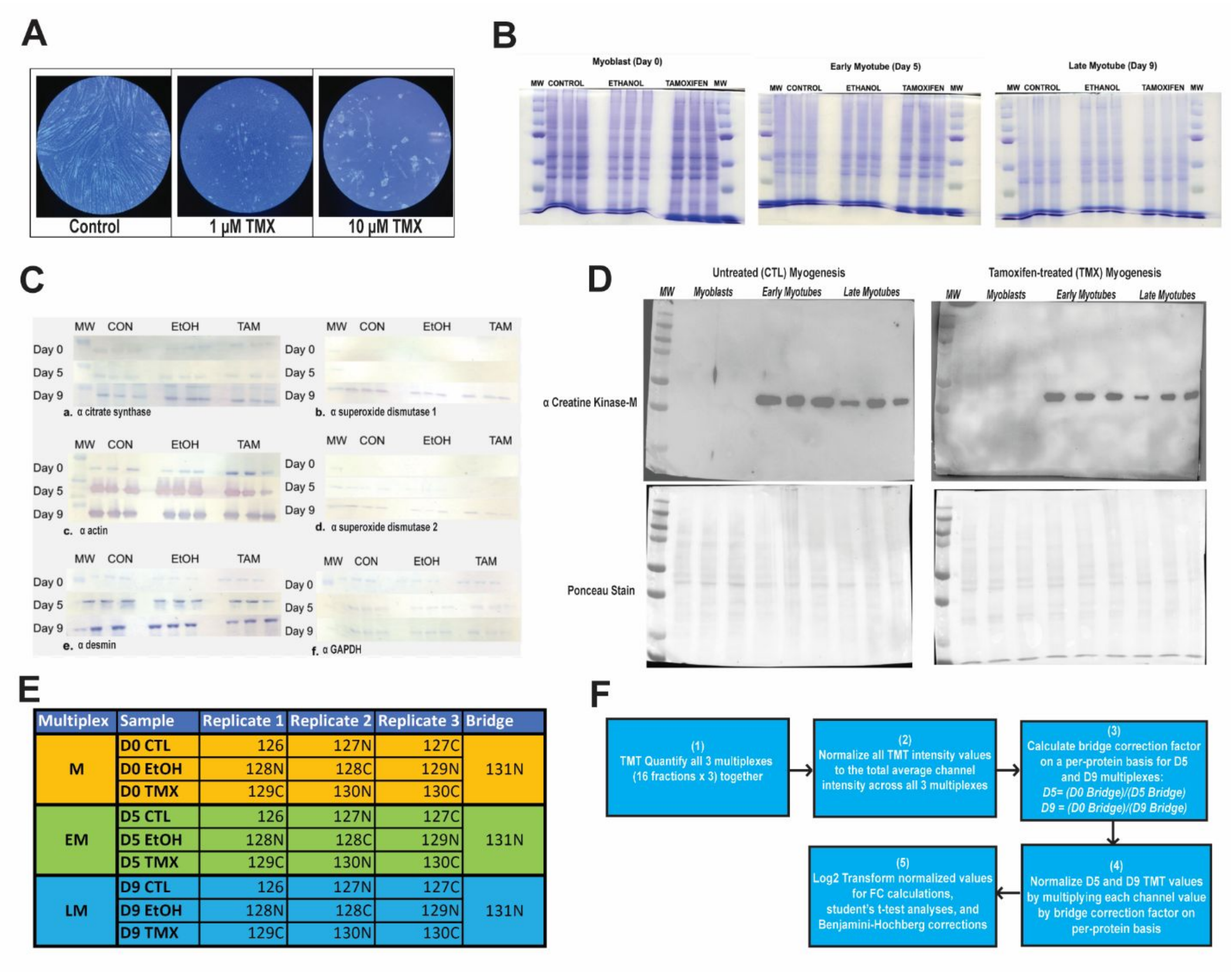

Figure S1: Tamoxifen dose optimization, protein gels, confirmation blots, and TMT schemes. (A) Representative images of C_2C_{12} cells grown in different TMX doses for 7 days. Control cells formed myotubes - the long, cylindrical structures; cells grown in 1 µM TAM did not fuse, and only one myotube was observed in the field of view; cells grown in 10 µM TAM showed signs of cytotoxicity as the cells were less numerous, and there were no signs of myotube formation. **(B)** 1D-SDS PAGE gels were stained with Coomassie Brilliant Blue R250 to compare total protein levels across different treatment conditions. **(C)** Colorimetric assay immunoblots measuring five common markers of myogenesis (citrate synthase, superoxide dismutase 1, alpha-actin, superoxide dismutase 2, and desmin; GAPDH was used as a loading control) across all three treatments and time points; the same samples were used for the corresponding downstream TMT analysis. Band intensities correspond to expected literature values for each protein as a function of myogenesis, and no significant differences were detected by Image-J AUC analysis between treatment conditions (not shown). **(D)** Uncropped membranes of chemiluminescent anti-Creatine Kinase-M blots shown in Figure 1B and the corresponding Ponceau stains; 20 µg of protein were loaded per lane as determined by Lowry assay. EtOH (vehicle) is not shown but

the expression pattern was identical to untreated and TMX+vehicle; the same samples were used for TMT analysis (as well as for the blots shown in (C)). **(E)** TMT-labeling scheme for the three multiplexes shown in Figure 1C. The same aliquots of TMT-11plex reagents were used to label 40 µg of peptide per sample from each multiplex. The bridge channel was generated by combining 15 µg of peptide from each sample in the Replicate #1 column, resuspending in 135 µL of EPPs buffer, and dividing the combined sample into 3 x 40 µg (40 µL each, with 15 µL left over) before labeling in triplicate with the same aliquot of TMT-11plex reagent (channel 131N). **(F)** TMT normalization scheme. TMT results from all 16 fractions of each multiplex (48 total) were quantified as a group using in-house software. All TMT intensity values were then normalized to the total, average channel intensity of all three multiplexes. A bridge-correction factor was calculated per-protein as described to normalize the Day 5 (Early Myotube; EM) and Day 9 (Late Myotube; LM) multiplexes to the Day 0 (Myoblast) multiplex, and bridge-corrected values were log2 transformed to ensure a normal distribution. Fold change calculations and student's t-test were performed on the normalized, corrected, and transformed TMT values.

A

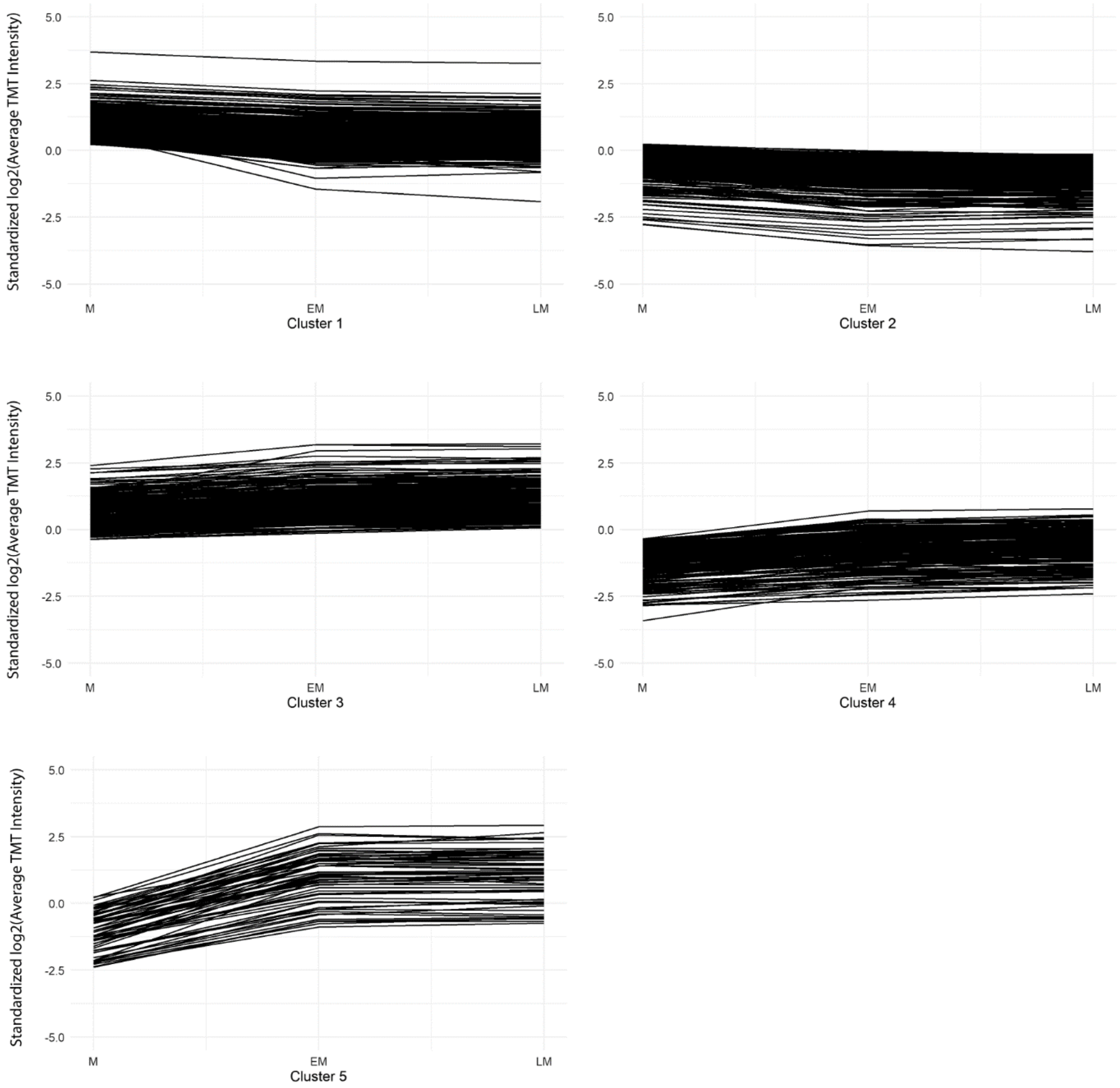

B-F: SVG Files Included as supplemental data*

Figure S2: Full cluster analysis of myogenically regulated proteins in the untreated longitudinal analysis. (A) Standardized (median=0, std= 1), average $\log_2$ TMT values for each cluster were plotted to show actual trajectories (rather than FC to Day 0 trajectories as in Figure 2). **(B-F)*** Full networks generated by STRING-db shown in Figure 2B for each MR cluster in the untreated myogenesis dataset; corresponding enrichment analysis for each network can be found in Supplemental Table 5.

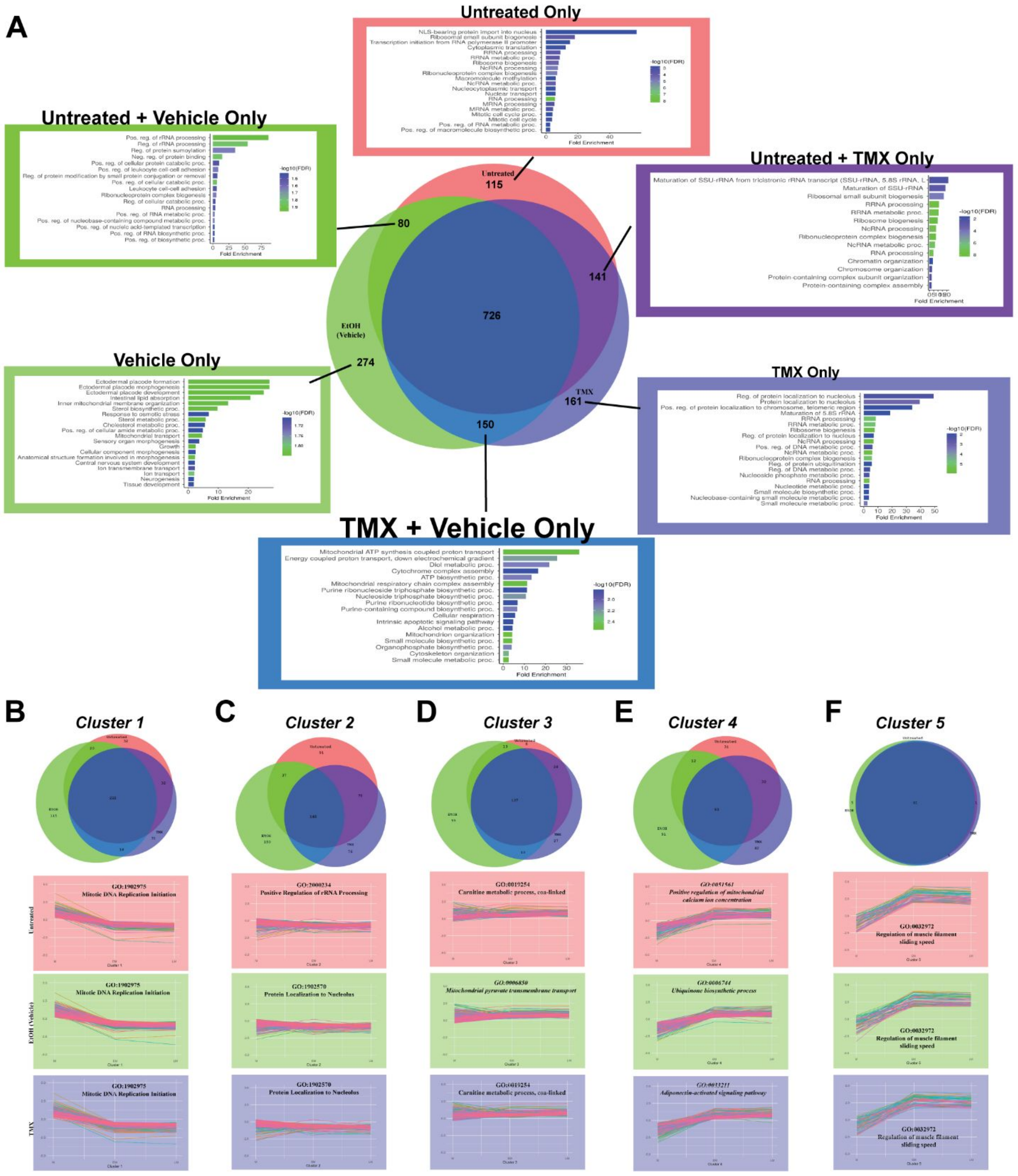

Figure S3: Comparison of uncorrected longitudinal clustering analysis between untreated, EtOH vehicle-treated, and vehicle+TMX-treated myogenesis. (A) Overlaps of all MR proteins from the untreated, EtOH-treated, and TMX-treated analyses.[49] EtOH alone had a distinct effect on the myogenic program, but the overlapping (TMX+Vehicle Only; n=150) proteins were

removed from the final longitudinal TMX analysis shown in Figure 3. GO:BP enrichments were performed on each overlap using ShinyGO.[20] "Untreated Only" proteins (n=115) were enriched for ribosome biogenesis and mRNA regulation; "Untreated+Vehicle Only" proteins (n=141) were enriched for rRNA processing and mitochondrial ion transport. "Vehicle Only" proteins (n=274) primarily enriched for apoptotic processes associated with embryogenesis, as well as other stress-related processes. "TMX+Vehicle Only" proteins (n=150) were only enriched for the intrinsic apoptotic signaling pathway, suggesting a small but potentially detrimental interaction of EtOH and TMX in C_2C_{12} cells. "TMX Only" proteins (n=161) were enriched for telomere localization and maintenance (discussed in section 3 of the main body of this study). Finally, "Untreated + TMX Only" proteins were enriched for protein localization to the nucleolus and rRNA processing, similar to the enrichment found in cluster 2 of both the untreated and TMX longitudinal analysis in Figure 3. **(B-F)** Overlaps by individual cluster from the untreated, EtOH, and (uncorrected) TMX longitudinal analyses. EtOH's effect on MR is noticeably clearer at the cluster level, but the top GO:BP terms remained unchanged between all three treatment conditions for clusters 1, 2, and 5.[49] Interestingly, the top GO:BP terms for cluster 3, while unchanged between TMX and untreated MR analyses, was different for EtOH cluster 3; however, the primary functional enrichment of this cluster was still related to small molecule and energy metabolism. Cluster 4 was the only cluster that was distinct in functional enrichment between all 3 treatment conditions, and in EtOH myogenesis cluster 4 shifted from mitochondrial regulation in the untreated analysis to "muscle fiber development". The expression trend of cluster 4 was the same across all three treatments: a modest fold change increase from myoblasts to late myotubes. While cluster 5 (skeletal muscle myogenesis-specific proteins) was essentially unchanged, the enrichment for ubiquinone biosynthetic processing & muscle fiber development (ST6) in EtOH cluster 4 suggests a potential change in the rate of overall muscle development and protein turnover, which is supported by other EtOH-Myogenesis studies in the literature.[22] However, further analysis is required to fully understand the implications of EtOH-treated myogenesis. Full protein lists by cluster from each treatment condition can be found along with complete corresponding functional enrichments in Supplemental Tables 2,4 & 6.

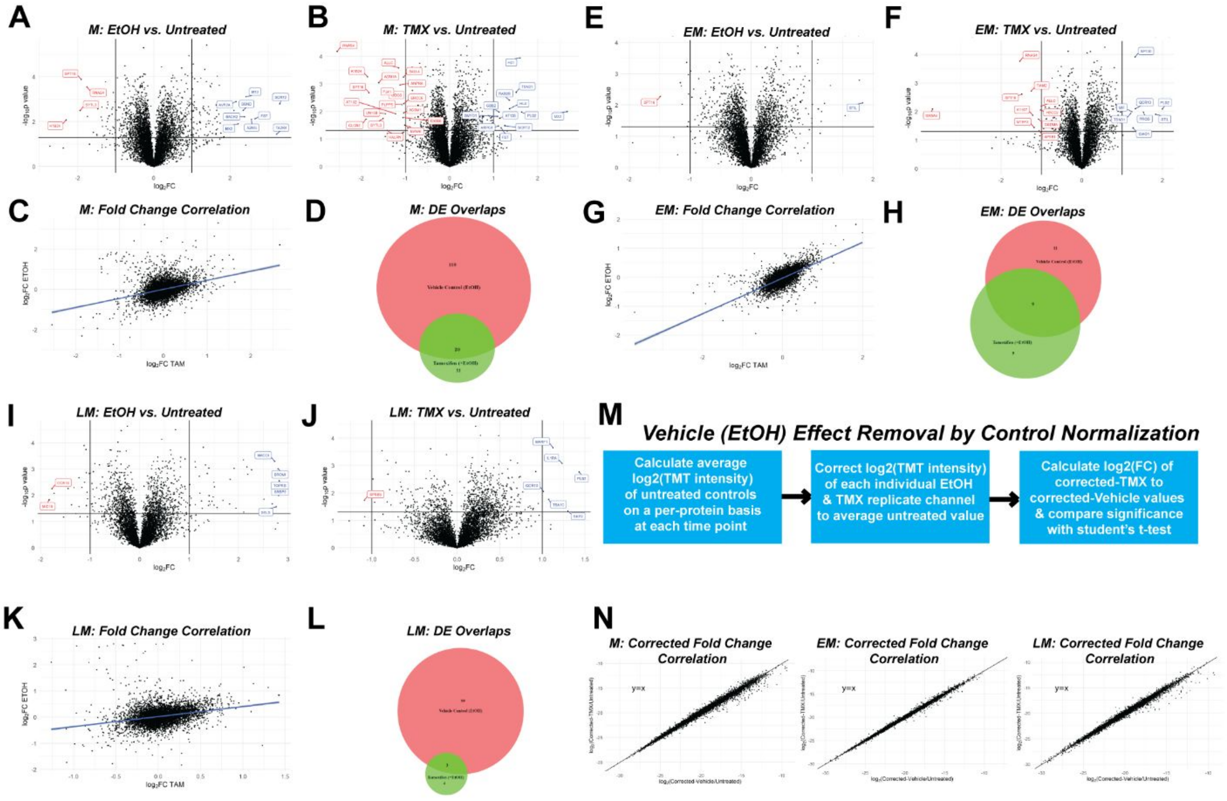

Figure S4: The effect of EtOH (Vehicle) on differential expression at each time point & vehicle-effect removal scheme. (A) Volcano plot comparing EtOH-induced differential expression of proteins relative to untreated controls in myoblasts; top 10% of FC are labeled. **(B)** Volcano plot comparing uncorrected, TMX-induced (i.e., TMX+vehicle-induced) differential expression of proteins relative to untreated controls in myoblasts; all proteins are labeled. **(C)** A correlation plot comparing $\log_2$(FC) values from EtOH/Untreated to TMX/Untreated. **(D)** Total overlap of uncorrected DE proteins from C.[49] **(E-L)** are identical to **(A-D)** but show the DE proteins from Early Myotubes **(E-H)** and Late Myotubes **(I-J)**. These comparisons revealed a striking effect on DE proteins at each time point as a result of vehicle treatment; this is most evident in the correlation plots, which (if the vehicle had no effect) should have a 1:1 correlation with only TMX-induced DE proteins falling out of the y=x slope. To remove the effect of the vehicle from DE analysis of TMX, TMT values at each time point were normalized to the untreated myogenesis values as described in **M**. **(N)** Corrected fold change comparisons now show a 1:1 correlation, with proteins specific to TMX-treatment falling off of the y=x line. Comparisons between corrected-TMX and corrected-Vehicle (Figure 4) account for the majority of vehicle effect by normalizing both treatments to a baseline value and by "subtracting" vehicle effect from the TMX condition via $\log_2$(FC). Based on the corrected correlation plots, the overlap of our results from the DE vs. MR protein analysis shown in Figure 5, and prior literature studies on TMX-based regulation, we are confident in the accuracy of the data presented. However, from the results shown here, we suggest that future studies of TMX (or other drugs) on

myogenesis make use of a different vehicle (such as DMSO) wherever possible so as to avoid potential EtOH-drug interactions in proteomic data, *etc*.

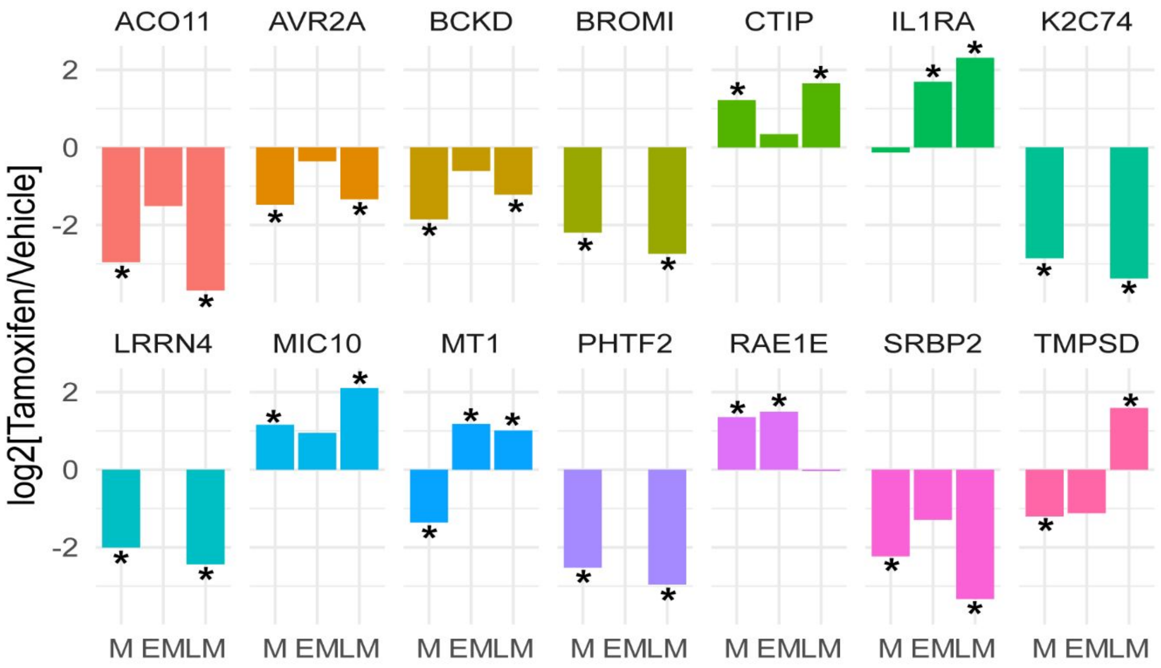

Figure S5: All proteins differentially expressed by TMX treatment at 2 or more time points. While 10 proteins were detected at all 3 stages of myogenesis and DE (FC ≥ |2|, BH-adjusted $p < 0.05$ by student's t-test) at 2 or more stages, another 4 proteins (BROMI, K2C74, LRRN4, and PHTF2) were DE expressed at 2 time points but not detected at the third. Interestingly, the lack of detection occurred in the early myotube multiplex for all 4 of these proteins, despite the presence of a bridge channel. As MS3-TMT analysis requires a certain initial signal threshold in the MS2 scan in order for the mass spectrometer to trigger a third, high energy fractionation of the isobaric tags, the presence of a particular peptide in only a few channels out of ten often results in too low of a signal for sequencing/quantification scans. We suspect that these five proteins were not sequenced in the early myotube multiplex as a result of low signal in three or more of the channels. However, their significance at the remaining time points suggests they warrant follow-up study in TMX-myogenesis models. A full list of all highly regulated proteins from this figure with their corresponding fold changes, eggNOG designations, and any available literature referencing TMX can be found in Table S9.
